# Adipose Tissue Remodeling Beyond Weight Loss: New Insights Into Incretin-Based Therapies and Bariatric Surgery

**DOI:** 10.1007/s13679-026-00756-x

**Published:** 2026-09-10

**Authors:** Gabriela Ferreira Abud, Ellen Cristini de Freitas, Kristin I. Stanford

**Affiliations:** 1 Dorothy M. Davis Heart and Lung Research Institute, The Ohio State University Wexner Medical Center, 460 West 12th Ave. 314 Biomedical Research Tower, Columbus, OH 43210 USA; 2 Department of Surgery, Division of General and Gastrointestinal Surgery, The Ohio State University Wexner Medical Center, Columbus, OH USA; 3 Department of Health Sciences, Ribeirao Preto Medical School, University of Sao Paulo, USP, Ribeirao Preto, Sao Paulo, Brazil; 4 School of Physical Education and Sport of Ribeirao Preto (EEFERP/USP), University of Sao Paulo, Ribeirao Preto, Brazil

**Keywords:** Obesity, White adipose tissue, Incretins, Bariatric surgery, Molecular mechanisms

## Abstract

**Purpose of the Review:**

This review summarizes obesity-induced adaptations in subcutaneous and visceral white adipose tissue (WAT) depots and discusses how incretin-based pharmacotherapies and bariatric surgery may influence WAT remodeling beyond weight loss, drawing on evidence from animal models and human studies.

**Recent Findings:**

In obesity, adipose tissue undergoes pathological remodeling characterized by structural and cellular changes, extracellular matrix (ECM) remodeling, altered endocrine signaling, and impaired mitochondrial function that contribute to metabolic comorbidities. Weight-loss interventions, including incretin-based therapies and bariatric surgery, are associated with improvements in adiposity and metabolic outcomes, and multiple studies suggest accompanying changes in inflammatory, mitochondrial/thermogenic, and endocrine pathways within WAT; however, mechanistic evidence in humans remains heterogeneous and is often limited by tissue accessibility and study design.

**Summary:**

This review highlights candidate cellular and molecular mechanisms through which incretin-based pharmacotherapies and bariatric surgery may modulate WAT plasticity, emphasizing convergent and distinct effects across depots and the need for more longitudinal, tissue-level human studies to define weight-dependent remodeling versus changes not fully explained by weight loss.

## Introduction

Obesity is a chronic, multifactorial disease that has reached epidemic proportions, currently affecting approximately 800 million individuals worldwide [1]. In this condition, alterations in adipose tissue contribute to comorbidities such as cardiovascular disease (CVD), type 2 diabetes mellitus (T2D), and arterial hypertension (AH), among others [2, 3]. Adipose tissue undergoes pathological remodeling characterized by alterations in cellular composition, pro-inflammatory signaling pathways, extracellular matrix (ECM) composition, vascularization, and mitochondrial function [4]. A range of therapeutic strategies, including incretin-based therapies and bariatric surgery, have been used to promote weight loss and mitigate obesity-related complications [3, 5]. In this review, we focus on how these therapeutic strategies influence white adipose tissue (WAT) remodeling, including potential effects that extend beyond weight loss. Where available, we distinguish responses in subcutaneous (scWAT) and visceral (vWAT) WAT depots, while noting that many human studies are limited by preferential sampling of scWAT and sparse paired-depot analyses.

This narrative review provides an overview of obesity-induced changes in adipose tissue, focusing on scWAT and vWAT depots, including inflammation, mitochondrial function, and endocrine activity. We then synthesize evidence on how incretin-based therapies and bariatric surgery are associated with WAT remodeling, emphasizing proposed molecular pathways and highlighting where evidence supports weight-independent effects and where changes are not fully explained by reduced adiposity. Finally, we discuss the magnitude of weight loss, long-term management, health outcomes, and the emerging role of incretin-based pharmacotherapy following bariatric surgery.

Throughout this review, we use the term “beyond weight loss” to denote adipose tissue adaptations that cannot be fully explained by reductions in fat mass or negative energy balance alone. Operationally, evidence for weight-independent remodeling is strongest when studies include weight-matched comparators (e.g., pair-fed or caloric restriction controls), statistical adjustment or mediation analyses showing persistence of tissue-level effects after accounting for weight change, or temporal dissociation in which molecular or cellular changes in WAT precede substantial weight loss. In humans, where such controls are often not feasible, we interpret “beyond weight loss” more cautiously as changes that persist when adiposity is comparable, are observed with localized or ex vivo exposure, or are consistent with receptor/pathway engagement within adipose-resident cell populations. This framework is intended to distinguish direct or tissue-autonomous actions from secondary consequences of improved systemic metabolism, reduced adipocyte hypertrophy, and altered nutrient flux that accompany weight reduction.

## Obesity-Induced Alterations in Adipose Tissue

Adipose tissue is a complex connective tissue composed of mature adipocytes and a stromal vascular fraction (SVF) that includes preadipocytes, fibroblasts, endothelial cells, macrophages, T and B lymphocytes, and other immune cells [4, 6]. It is a highly dynamic, metabolically active tissue capable of extensive remodeling in response to physiological and environmental stimuli [4]. Adipose tissue is broadly classified into white adipose tissue (WAT), brown adipose tissue (BAT), and beige adipose tissue, also referred to as brite adipose tissue [7]. WAT is composed of unilocular adipocytes that store energy in the form of triglycerides (TGs) under anabolic conditions. WAT can be further subdivided into two anatomically distinct depots: subcutaneous WAT (scWAT), located beneath the skin, and visceral WAT (vWAT), also known as the omental fat depot, situated intra-abdominally adjacent to major organs in humans [7, 8].

BAT is predominantly located in the cervical, supraclavicular, and paravertebral regions in adult humans, and in the interscapular and perirenal regions in rodents and neonates [9, 10]. BAT is characterized by multilocular lipid droplets and abundant mitochondria, which support energy expenditure through heat dissipation mediated by uncoupling protein-1 (UCP-1), a thermogenic protein located in the inner mitochondrial membrane [9]. Beige adipose tissue is characterized by multilocular lipid droplets and can arise through remodeling of white adipocytes or recruitment of beige adipocyte programs within WAT, sharing morphological and functional similarities with brown adipocytes, including elevated thermogenic capacity [11]. In this review, we focus predominantly on how obesity and subsequent interventions are associated with changes in WAT.

## Inflammation of WAT in Obesity

In conditions such as obesity, WAT expansion and pathological remodeling are characterized by adipocyte hypertrophy (increase in cell size) and hyperplasia (increase in cell number) [4, 12]. Mature adipocytes develop from adipose progenitors (APs) located in the SVF of WAT and do not undergo cell division; rather, adipocyte hyperplasia is mediated through *de novo* adipogenesis, a process by which new adipocytes are formed from adipocyte precursor cells (APCs) into mature adipocytes [4, 12].

Obesity is associated with low-grade WAT inflammation, marked by infiltration of immune cells, including CD4⁺ T_H_1 and CD8⁺ T cells that secrete pro-inflammatory cytokines such as interferon-gamma (IFN-γ). IFN-γ promotes macrophage polarization from an anti-inflammatory to a pro-inflammatory profile and increases production of cytokines, including tumor necrosis factor-alpha (TNF-α), interleukin-6 (IL-6), and interleukin-1 beta (IL-1β) [12–14]. Evidence from animal models [15] and human clinical trials [16] in obesity has demonstrated the presence of both CD4⁺ and CD8⁺ T lymphocytes, along with macrophage accumulation, within adipose tissue.

Additionally, pro-inflammatory mediators contribute to pathological ECM remodeling within adipose tissue [17]. Adipocytes are surrounded by an ECM comprising a diverse network of fibrous proteins, including collagens, adhesion molecules, proteoglycans, and matrix-modulating enzymes such as matrix metalloproteinases (MMPs). These proteins provide structural support and regulate biological processes such as cell adhesion, migration, proliferation, and differentiation [17, 18]. In obesity, ECM components accumulate, remodel, and reorganize to accommodate adipocyte expansion. ECM accumulation is orchestrated by a balance between synthesis and assembly of matrix components, such as collagens and the procollagen C-endopeptidase enhancer (PCOLCE), and degradation of existing ECM by MMPs and their endogenous inhibitors, known as tissue inhibitors of matrix metalloproteinases (TIMPs) [17]. Among TIMP isoforms, TIMP3 appears to be reduced in obesity and insulin resistance and is associated with an exacerbated inflammatory response [19], although the available evidence remains limited.

## Mitochondrial Dysfunction of WAT in Obesity

In adipocytes, mitochondria regulate key processes such as glucose and lipid homeostasis, as well as adenosine triphosphate (ATP) production through the oxidative phosphorylation system (OXPHOS) located in the inner mitochondrial membrane [20, 21]. Obesity is associated with mitochondrial dysfunction in adipocytes, including decreased ATP production and mitochondrial respiration, and with increased reactive oxygen species (ROS), which are by-products of mitochondrial OXPHOS [21–24].

Studies in rodent models fed a high-fat diet (HFD) have demonstrated decreased mitochondrial ATP production in vWAT, along with suppression of peroxisome proliferator-activated receptor gamma coactivator-1 alpha (PGC-1α), the master regulator of mitochondrial biogenesis; PGC-1β; and the transcription factor peroxisome proliferator-activated receptor alpha (PPARα), which are involved in adipocyte lipid oxidation pathways, mitochondrial biogenesis, and thermogenic activity in adipose tissue [25, 26]. Similarly, another study using 12-week-old experimental models of obesity and diabetes reported reduced expression of mitochondrial proteins in adipocytes, including ATP synthase α and β subunits, as well as OXPHOS complex II and III components [27].

In humans with obesity, adipocyte hypertrophy has been proposed as a key factor driving mitochondrial dysfunction under caloric surplus [28]. Multiple regulators of mitochondrial biogenesis in WAT, including PGC-1α, along with mitochondrial DNA (mtDNA) content, mtDNA-encoded transcripts, and OXPHOS protein levels are downregulated in obesity [21, 29], and obesity has been associated with impaired activities of mitochondrial respiratory chain complexes in WAT, reduced ATP synthesis, and increased ROS generation [30, 31]. Another study in humans investigating oxidative stress-related enzymes in scWAT mitochondria from individuals with obesity or T2D reported increased mitochondrial ROS production, accompanied by elevated malondialdehyde (MDA) levels, and reduced activities of the antioxidant enzymes glutathione peroxidase (GPx) and superoxide dismutase (SOD) compared with the control group [22]. Overall, mitochondrial function plays a central role in adipose tissue biology, including cellular energy production, lipid metabolism, redox homeostasis, thermogenesis, and endocrine signaling, and warrants further investigation in the context of obesity.

## Endocrine Dysfunction of WAT in Obesity

WAT is an endocrine organ that secretes a wide range of adipokines, which play crucial roles in regulating energy storage and expenditure, glucose and lipid metabolism, inflammatory responses, and insulin sensitivity [32]. These adipokines act through autocrine, paracrine, and endocrine pathways, allowing adipocytes to modulate metabolic processes in adipose tissue and other organs and systems [33, 34]. Here, we discuss key adipokines secreted by WAT, including leptin, adiponectin, and retinol-binding protein-4 (RBP4), focusing on obesity-driven dysregulation of their physiological signaling [32].

### Leptin

 Leptin is a major adipokine produced by WAT that plays a critical role in regulating food intake and energy expenditure through activation of the leptin receptor (LepR), which is expressed in multiple organs, including the hypothalamus [35, 36]. Circulating leptin concentrations and leptin expression in adipose tissue are associated with adiposity and body weight changes [35, 37, 38]. In rodents and humans, circulating leptin levels are proportional to fat mass and elevated in obesity [37, 38]. Hyperleptinemia protects non-adipose tissues such as muscle, liver, and heart from lipotoxicity. However, when hyperleptinemia fails to maintain lipid homeostasis, lipotoxicity occurs in peripheral cells [39]. Additional evidence from human studies further supports these findings, demonstrating that weight gain is associated with increases in leptin levels and that obesity is linked to impaired leptin signaling [40].

### Adiponectin

Adiponectin, also known as ADIPOQ, is one of the most abundant adipokines expressed exclusively in adipocytes [41, 42]. Its circulating levels are reduced in the context of obesity, insulin resistance and T2D [42–44]. In HFD-induced models of obesity and altered insulin sensitivity, decreased adiponectin expression consistently correlates with insulin resistance [42, 43].

In humans, adiponectin levels are reduced in obesity, both at the circulating level and in WAT gene expression, and are inversely associated with visceral and subcutaneous adiposity and inflammatory markers such as TNF-α, collectively suggesting a link between adiponectin suppression, adipose tissue inflammation, and metabolic dysfunction [45–48].

### RBP4

 Another adipokine expressed in WAT is RBP4, although the liver is the main source of circulating RBP4. Elevated RBP4 has been associated with reduced insulin sensitivity, altered glucose homeostasis, inflammatory responses, and impaired adipose tissue function, as observed in both animal models and human studies [49–53]. Adipose-specific GLUT4 deficiency in rodents was associated with insulin resistance and elevated circulating RBP4 levels, suggesting that RBP4 may serve as a signal connecting adipose glucose uptake to systemic insulin sensitivity and that increased RBP4 may contribute to impaired insulin signaling [49].

In humans with obesity, RBP4 gene expression in scWAT is significantly reduced, but serum RBP4 levels do not differ among lean, overweight, and obese individuals. Notably, RBP4 and GLUT4 mRNA levels are positively correlated in scWAT [51]. This finding is consistent with other reports that RBP4 expression in scWAT correlates positively with GLUT4 expression [54], suggesting a link between adipose glucose uptake, GLUT4 expression, and regulation of RBP4 [51]. In individuals undergoing abdominal surgery, including laparoscopic or laparotomic cholecystectomy, hysterectomy, or gastric banding, RBP4 expression was lower in vWAT than in scWAT, suggesting that RBP4 may be less involved in glucose metabolism or insulin action in vWAT. However, no differences were observed in RBP4 mRNA expression between depots or in circulating RBP4 levels among lean individuals, individuals with obesity, or individuals with obesity and metabolic syndrome [53].

## Incretin-Based Therapies and WAT Remodeling

Incretins are gut hormones that enhance insulin secretion in response to nutrient intake. Notably, endogenous incretins stimulate glucose-dependent insulin release by pancreatic β-cells while also reducing glucagon secretion and slowing gastric emptying, thereby contributing to satiety and body weight regulation. In patients with obesity, however, fasting and postprandial circulating GLP-1 and GIP concentrations vary across studies and are not consistently lower than those observed in individuals without obesity [55, 56]. Thus, impaired incretin action in obesity or T2D should not be interpreted as uniformly reduced circulating GLP-1 or GIP concentrations.

GLP-1 is an incretin primarily produced by enteroendocrine L cells, as well as by specific neurons and a subset of pancreatic α-cells in the islets of Langerhans; it interacts with the GLP-1 receptor (GLP-1R). GIP is an incretin secreted by intestinal K cells and exerts its effects via the GIP receptor (GIPR), which is expressed in pancreatic β-cells as well as in other tissues and organs [5, 57]. GLP-1R and GIPR signaling pathways contribute to systemic metabolic regulation and may influence adipose tissue function. GLP-1R may predominantly improve systemic metabolic parameters, including insulin sensitivity and lipid metabolism, whereas GIPR signaling has been proposed to exert context-dependent effects within adipose tissue, including potential modulation of vascularization, lipid metabolism, inflammatory responses, and insulin-related signaling pathways [5, 58, 59]. However, the relevant adipose-resident target cell populations and the physiological significance of these effects remain incompletely understood.

Recent pharmaceutical interventions have focused on incretin-based therapies, including GLP-1R agonists such as liraglutide and semaglutide, as well as tirzepatide, a novel long-acting dual GLP-1/GIP receptor agonist [60–64]. Incretin-based therapies were initially approved by the Food and Drug Administration (FDA) for the treatment of T2D and later for the treatment of obesity [65].

Body weight regulation is orchestrated by complex interactions between the brain and adipose tissue, which communicate through signaling networks involving adipokines such as leptin and adiponectin [66, 67]. In response to adipokine signaling, the brain integrates these signals and regulates peripheral metabolic adaptations, including increased fatty acid oxidation, reduced plasma glucose levels, and regulation of energy expenditure, thereby contributing to changes in adipose tissue mass. These processes influence both the functional and structural characteristics of WAT [66]. Given the efficacy of incretin-based therapies in reducing body weight and improving metabolic homeostasis, part of their metabolic effects may be associated with altered adipokine signaling, although whether these changes arise directly within WAT or secondary to reduced adiposity remains unclear [67].

Although a growing body of evidence supports their efficacy in promoting substantial weight loss and improving obesity-related comorbidities, including T2D [61, 63, 64], some effects of these therapies may extend beyond reductions in body fat, although the extent to which they reflect direct adipose actions remains uncertain. These pharmacological strategies may influence adipose tissue through indirect mechanisms and, in some contexts, potential direct or local tissue-level effects, with possible implications for WAT phenotype and systemic metabolism [67]. These effects and their relevance to WAT remodeling are discussed in the following sections; however, the relative contributions of direct and indirect mechanisms remain insufficiently understood. This framework aligns with recent conceptual models positioning adipose tissue as an integrative mechanistic target of incretin- and glucagon-based anti-obesity agents, while emphasizing that the relative importance of direct adipose actions versus indirect systemic effects remains unresolved [67].

Given these gaps, the following sections summarize current evidence for GLP-1R agonists, dual GLP-1/GIP receptor agonists, and emerging triple GLP-1/GIP/glucagon receptor agonists in relation to WAT remodeling, with emphasis on inflammatory, mitochondrial/thermogenic, and endocrine pathways. Clarifying whether these effects are truly weight-independent or are better interpreted as changes not fully explained by weight loss remains a major limitation in the current literature.

## GLP-1R Agonists: Signaling and Intracellular Pathways

GLP-1R expression has been reported in both scWAT and vWAT depots in humans [68, 69]. Early studies reported GLP-1R in isolated human adipocytes [70], while subsequent experimental studies detected GLP-1R in differentiated 3T3-L1 adipocytes [71]. However, reliable detection of GLP-1R at both the tissue and cellular levels remains technically challenging, largely due to the limited availability of validated antibodies, which may contribute to uncertainty regarding GLP-1R abundance in adipose tissue and inconsistent findings across studies [72]. Reported GLP-1R expression also varies across species, adipose depots, and experimental systems, further limiting direct extrapolation from rodent or cell-model studies to mature human adipocytes in vivo. In addition, GLP-1R detected in bulk WAT samples may originate, at least in part, from non-adipocyte cell populations within the tissue, consistent with reports that GLP-1R expression in human vWAT is enriched within the SVF, which includes mesenchymal stem cells, preadipocytes, fibroblasts, vascular endothelial cells, and immune cells [69]. Therefore, although GLP-1R expression has been reported in adipose tissue, its abundance in mature adipocytes, physiological relevance, and relative contribution to direct adipocyte actions versus indirect systemic mechanisms remain incompletely understood [67].

Most mechanistic understanding of GLP-1R intracellular signaling derives from pancreatic β-cell and cell-model studies. Endogenous GLP-1 and GLP-1R agonists signal through GLP-1R, a G protein-coupled receptor (GPCR), and activate pathways involving Gα_s_-containing heterotrimeric G-proteins, cyclic AMP (cAMP), protein kinase A (PKA), β-arrestin 1, extracellular signal-regulated kinases (ERK1/2), and protein kinase B (PKB, also known as Akt) [73–76]. These pathways regulate cellular processes such as growth, differentiation, proliferation, and apoptosis, and may be relevant to adipocyte biology. In addition to ERK signaling, the protein kinase C (PKC) family regulates proliferation and apoptosis through MAPK-ERK-dependent signaling and appears to contribute to adipogenesis [75, 77, 78]. In contrast, reduced GLP-1R expression may impair adipogenic processes and promote apoptosis [75].

## Effects of GLP-1R Agonists on WAT Remodeling: Modulation of Inflammatory, Mitochondrial, Thermogenic, and Endocrine Pathways

### Modulation of WAT Inflammation

Multiple studies suggest that GLP-1R agonists may modulate inflammatory pathways in WAT. However, evidence supporting direct anti-inflammatory effects on adipocytes or adipose-resident cells, independent of changes in adiposity and whole-body metabolism, remains limited. Instead, available evidence from animal and clinical studies indicates that improvements in WAT inflammation occur concomitantly with reductions in adiposity and improvements in metabolic health [79–82].

In HFD-induced obese rodents, 17 days of liraglutide treatment increased preadipocyte differentiation-related responses [75]. Another study demonstrated that 4 weeks of semaglutide treatment significantly reduced fat mass (vWAT, -55%; scWAT, -40%), expression of pro-inflammatory genes, including TNF-α, IL-6, and IL-1β, and mitigated adipocyte hypertrophy and macrophage infiltration in vWAT [79]. Together, these rodent studies suggest that GLP-1R agonist treatment may attenuate inflammation within WAT, although these changes occurred in parallel with substantial reductions in adiposity.

In human studies, clinical trials have investigated whether GLP-1R agonists are associated with remodeling of scWAT or vWAT [80, 82]. In adults who are overweight or have type 2 diabetes, liraglutide administered at 1.2 mg/day for 16 weeks induced 3.95 kg of weight loss and reduced BMI [82]. In individuals with obesity, liraglutide treatment at 1.8 mg/day for 26 weeks resulted in 2.4 kg of weight loss, accompanied by reduced BMI and visceral fat and improved glucose metabolism [80]. Despite these metabolic improvements, no suppression of pro-inflammatory pathways, change in adipocyte size, or induction of ECM remodeling was observed in scWAT [80, 82].

Additional evidence from human adipose tissue was obtained using epicardial adipose tissue (EAT). Semaglutide treatment (0.5 or 1.0 mg once weekly for 24 weeks) induced significant weight loss (9%) and altered the protein cargo of EAT-derived exosomes, attenuated pro-inflammatory signaling ex vivo, and reduced circulating levels of the adiposity marker fatty acid-binding protein 4 (FABP4) [83], an adipokine expressed in adipocytes and macrophages that plays an important role in insulin resistance [84]. Although these findings suggest local changes within adipose tissue, they do not establish whether these responses result from direct GLP-1R signaling or are secondary to systemic metabolic improvements.

Collectively, current evidence suggests that GLP-1R agonists are associated with improved systemic metabolic health and reduced adiposity. Animal studies support attenuation of inflammatory pathways within WAT, whereas clinical studies have demonstrated more heterogeneous tissue-level responses despite significant weight loss and metabolic improvement. Therefore, improvements in WAT inflammation observed in clinical studies may be mediated, at least in part, through indirect mechanisms driven by central regulation of appetite, reduced energy intake, negative energy balance, and subsequent weight loss. Although GLP-1R agonists may influence inflammatory remodeling of WAT, it remains unclear whether these effects represent direct adipose tissue actions beyond weight loss or secondary consequences of reduced adiposity and improved whole-body metabolism [67].

### Mitochondrial Remodeling and Potential Thermogenic Modulation of WAT

Evidence from in vitro adipocyte models and rodent studies suggests that GLP-1R agonists may modulate markers associated with mitochondrial biogenesis and thermogenesis in scWAT and vWAT [79, 85–87]. In human SGBS adipocytes, an in vitro model derived from scWAT, liraglutide increased mitochondrial respiration and the expression of markers associated with mitochondrial biogenesis and a beige-like adipocyte phenotype, including PGC-1α, UCP-1, PRDM16, CIDEA, and transmembrane protein 26 (TMEM26). These findings provide in vitro mechanistic evidence that liraglutide can modulate mitochondrial and thermogenic-related pathways in this human adipocyte model [88].

Several studies in HFD-induced obese rodents reported that GLP-1R agonist treatment increased markers associated with mitochondrial biogenesis and thermogenic remodeling in scWAT and vWAT [79, 85–87]. Twelve weeks of liraglutide treatment increased expression of browning-associated genes, including cell death-inducing DFFA-like effector A (CIDEA), PPARγ, PR domain containing 16 (PRDM16), and UCP-1, as well as mitochondrial-related markers such as PGC-1α and mitochondrial transcription factor A (TFAM), in both scWAT and vWAT [87]. Similarly, shorter liraglutide or semaglutide interventions increased expression of UCP-1, PRDM16, PPARα, PGC-1α, NRF1, TFAM, and related AMPK/SIRT-1/PGC-1α signaling markers in scWAT or vWAT, although UCP-1 induction in visceral depots may be limited relative to subcutaneous depots [79, 85, 87, 89].

Human tissue-level evidence for GLP-1R agonist effects on mitochondrial and lipid-oxidation-related pathways in WAT remains limited. In a clinical trial investigating liraglutide treatment at 1.8 mg/day for 26 weeks in individuals with obesity and diabetes, treatment was associated with 3.9 kg of weight loss and upregulation of adipose tissue triglyceride lipase (ATGL) and carnitine palmitoyl transferase-1 (CPT-1) in scWAT [81]. ATGL promotes lipolysis of stored triglycerides, while CPT-1 facilitates the transport of free fatty acids released by lipolysis across the mitochondrial membrane into the mitochondrial matrix for β-oxidation [90–92]. Additionally, PPARα and PPARδ expression were increased, along with elevated UCP-2 expression [81]. However, because these molecular adaptations occurred concomitantly with substantial weight loss and metabolic improvement, it remains difficult to distinguish direct adipose tissue effects from secondary responses to reduced adiposity and improved systemic metabolism, and current human tissue-level evidence does not establish induction of beige adipocytes or functional WAT browning by GLP-1R agonists [93].

### Endocrine Modulation of WAT

Previous evidence indicates that GLP-1R agonists may modulate the endocrine function of WAT [79, 94–97]. An in vitro study reported that a GLP-1R agonist increased adiponectin secretion through activation of the protein kinase A pathway in differentiated 3T3-L1 adipocytes [96].

In rodent models, GLP-1R agonist treatment increased leptin receptor (LepR) expression and STAT3 phosphorylation in scWAT and vWAT, findings consistent with enhanced leptin-related signaling [94]. Across studies with treatment periods ranging from 4 to 20 weeks, liraglutide or semaglutide reduced circulating leptin and retinol-binding protein 4 (RBP-4) levels while increasing adiponectin expression [79, 95, 97], in addition to decreasing leptin expression in both scWAT and vWAT [79, 94].

Evidence in humans remains heterogeneous and is based largely on circulating adipokines, with limited tissue-level analyses. In individuals with overweight or obesity, liraglutide treatment at 1.2 mg/day for 16 weeks did not change leptin or adiponectin expression in scWAT, although circulating adiponectin increased in individuals who achieved weight loss [80]. In contrast, liraglutide at 1.8 mg/day for the same duration significantly reduced plasma leptin levels, while adiponectin levels remained unchanged [81]. These findings were accompanied by significant improvements in glycemic parameters [80, 81] and insulin sensitivity [81].

Other studies reported no changes in serum adiponectin or leptin concentrations after 26 weeks of liraglutide at doses between 1.2 and 1.8 mg/day [82], whereas semaglutide treatment at 0.5 or 1.0 mg once weekly for 6 months reduced plasma leptin levels [83]. However, these endocrine adaptations occurred concomitantly with weight loss and improvements in metabolic health, making it difficult to distinguish direct adipose tissue effects from secondary responses to reduced adiposity.

Overall, GLP-1R agonist effects on WAT endocrine function remain supported primarily by in vitro and rodent studies, whereas human evidence is heterogeneous and largely limited to circulating adipokines.

### Dual GLP-1/GIP Receptor Agonists: Signaling and Intracellular Pathways

The interpretation of WAT responses becomes more complex for dual GLP-1/GIP receptor agonists because GIPR signaling may involve adipose-resident and non-adipocyte cell populations differently from GLP-1R signaling. In adipocyte models, endogenous GLP-1 and GIP, as well as dual GLP-1/GIP receptor agonists, have been reported to activate intracellular signaling through GLP-1R and GIPR, including in differentiated 3T3-L1 adipocytes [98, 99]. Upon activation, GIPR, also a member of the GPCR superfamily, stimulates intracellular cAMP production through Gαs, while β-arrestin contributes to regulation of GIPR signaling [100–102]. Although adipocyte-model studies support the capacity of GIPR signaling to regulate adipocyte-relevant pathways, translating these findings to intact adipose tissue is complicated by uncertainty regarding GIPR localization.

While evidence suggests that GIPR is expressed in both the SVF and mature adipocyte fractions of scWAT in rodents and humans [99], the precise cellular localization and physiological role of GIPR in adipose tissue remain uncertain. Notably, Campbell et al. reported that single-nucleus RNA-sequencing adipose tissue datasets showed that Gipr/GIPR expression was predominantly localized to non-adipocyte cell populations, particularly pericytes and mesothelial cells, rather than mature adipocytes. Therefore, understanding GIP actions in specific GIPR-expressing adipose tissue cell populations remains limited, in part because of methodological challenges, including the lack of highly specific tools for GIPR detection [57]. In this context, the cellular localization and functional relevance of GIPR in adipose tissue remain controversial, and interpretations of tirzepatide-associated adipose tissue effects should not assume that mature adipocytes are the dominant target cell type.

Conversely, recent mouse studies using adipocyte-targeted GIPR manipulation suggest that adipocyte GIPR signaling can regulate thermogenic programs and futile calcium cycling in scWAT. These findings support the possibility of adipocyte-autonomous GIPR actions under experimentally induced or adipocyte-targeted conditions, but they do not resolve the broader controversy regarding endogenous GIPR localization and function in human WAT. Instead, the current literature suggests that GIPR biology in adipose tissue may depend on species, depot, metabolic state, and methodological approach.

Within this unresolved framework, GIP has been proposed to exert feeding status-dependent effects on adipose tissue metabolism, acting synergistically with insulin. In the fed state, GIPR activation may promote lipid storage by increasing lipoprotein lipase activity and enhancing glucose uptake in WAT. In contrast, during fasting, when insulin levels are low, GIP may favor lipolysis [5, 67].

Given this unresolved GIPR biology, interpretation of tirzepatide-associated adipose tissue remodeling requires caution, even as dual GLP-1/GIP receptor agonists have become a major focus in obesity treatment [99]. Tirzepatide, a dual GLP-1/GIP receptor agonist, induces effects that differ from those of endogenous GIP due to its extended duration of action, which persists beyond the postprandial period into the absorptive phase. Although tirzepatide is classified as a full agonist at both GLP-1R and GIPR, it acts as a biased agonist at GLP-1R, as it recruits β-arrestin to a lesser extent than endogenous GLP-1. This signaling profile favors cAMP generation over β-arrestin recruitment and is associated with a reduced capacity to induce GLP-1R internalization [102–104].

## Effects of Dual GLP-1/GIP Receptor Agonists on WAT Remodeling: Modulation of Inflammatory, Mitochondrial, Thermogenic, and Endocrine Pathways

### Modulation of WAT Inflammation

Evidence demonstrating direct anti-inflammatory effects of dual GLP-1/GIP receptor agonists on adipocytes or specific adipose-resident cells remains limited. However, recent animal studies suggest that dual GLP-1/GIP receptor agonists may modulate inflammatory pathways within adipose tissue [24, 105].

Notably, a study examining the anti-obesity effects of tirzepatide over 14 days in HFD-induced obesity models showed that tirzepatide attenuated macrophage accumulation in vWAT, as indicated by lower F4/80 expression and fewer crown-like structures (CLS). This reduction was accompanied by a shift in gene expression toward an anti-inflammatory profile, including decreased expression of adhesion G protein-coupled receptor E1 (Adgre1) and TNF-α, suggesting attenuated pro-inflammatory signaling [105].

In line with these findings, another study in HFD-induced obese rodents investigated the effects of tirzepatide over 12 weeks on adipose tissue inflammatory pathways. Tirzepatide was associated with markers consistent with macrophage apoptosis, altered ERK signaling, and attenuation of inflammatory cytokine levels, including TNF-α, IL-6, MCP-1, and IFN-γ, in vWAT. These effects were accompanied by reductions in both the size and weight of vWAT and scWAT in tirzepatide-treated animals [24]. Consistently, these studies also reported improvements in insulin sensitivity and reductions in body weight [24, 105].

In addition, preclinical evidence suggests that tirzepatide improves insulin sensitivity through both weight-dependent and weight-independent mechanisms. In obese insulin-resistant mice treated for 14 days, tirzepatide produced greater whole-body insulin sensitization than that observed in weight-matched semaglutide-treated or pair-fed groups. Comparison with these weight-matched groups suggested that approximately 30% of the total improvement in insulin sensitivity occurred independently of weight loss, supporting a GIPR-dependent component of this effect [106]. This study is particularly important for the “beyond weight loss” framework because it provides weight-matched preclinical evidence that at least part of tirzepatide’s insulin-sensitizing effect can be dissociated from reductions in body weight, although whether this effect reflects direct adipose tissue remodeling remains unresolved.

In humans, clinical evidence testing the hypothesis that tirzepatide modulates inflammatory markers within adipose tissue remains limited. A recent systematic review and meta-analysis reported that tirzepatide treatment was associated with significant reductions in circulating high-sensitivity C-reactive protein (hsCRP) and IL-6 levels. Specifically, once-weekly subcutaneous tirzepatide at doses of 5, 10, and 15 mg, compared with placebo, was associated with lower hsCRP and IL-6 concentrations. These findings suggest that tirzepatide is associated with systemic anti-inflammatory changes, likely driven by weight loss and improved metabolic parameters, including glycemic control and lipid profiles. However, because these markers were measured in circulation, they do not provide direct evidence of changes within adipose tissue. The authors emphasized important limitations and highlighted the need for studies directly evaluating adipose tissue responses to tirzepatide, including ex vivo studies and clinical trials capable of distinguishing direct adipose tissue effects from secondary consequences of weight loss [107].

Collectively, current evidence suggests that tirzepatide is associated with attenuation of obesity-related inflammatory pathways. Animal studies report tissue-level reductions in macrophage infiltration and inflammatory cytokine expression within WAT, whereas clinical evidence remains limited to circulating inflammatory biomarkers. Therefore, it remains unclear whether these anti-inflammatory adaptations represent direct adipose tissue effects beyond those attributable to weight loss and improved systemic metabolism. Moreover, given the unresolved cellular localization and functional relevance of GIPR in adipose tissue, these effects should be interpreted with caution. Future studies are needed to define the adipose-resident cell populations and indirect systemic mechanisms involved in inflammatory responses to dual GLP-1/GIP receptor agonists.

### Mitochondrial Remodeling and Potential Thermogenic Modulation of WAT

Evidence regarding the effects of dual GLP-1/GIP receptor agonists on mitochondrial metabolism and thermogenic remodeling of WAT remains limited [105, 108, 109]. A study examined lipolytic and thermogenic regulators in scWAT of HFD-induced obese rodents treated with tirzepatide for 14 days. Following the intervention, treated animals showed increased levels of phosphorylated hormone-sensitive lipase (P-HSL) and mitochondrial proteins, including phosphorylated cAMP response element–binding protein (P-CREB), UCP-1, and cytochrome c oxidase subunit 4 (COXIV) in scWAT, indicating changes in proteins associated with lipolysis, mitochondrial abundance, and thermogenic signaling [105].

However, animal studies have reported heterogeneous findings. In HFD-induced obese male rodents, localized administration of tirzepatide into scWAT for 4 weeks did not induce transcriptional activation of genes associated with mitochondrial biogenesis or thermogenic activity. Nevertheless, local treatment improved lipid metabolism and leptin signaling, while complementary ex vivo analyses of scWAT explants showed activation of the AMPK/ACC1 and JAK2/STAT3 pathways downstream of LepR signaling, indicating possible improved leptin responsiveness within adipose tissue [108]. Evidence in humans remains limited and does not yet provide an assessment of mitochondrial or thermogenic remodeling within WAT [109].

### Endocrine Modulation of WAT

Clinical studies have consistently reported increased circulating adiponectin levels [110, 111] and reduced leptin concentrations [110] after tirzepatide treatment, accompanied by improvements in insulin sensitivity [110, 111] and body weight [108–112]. However, because these observations are based primarily on circulating biomarkers, they do not establish whether dual GLP-1/GIP receptor agonists directly modulate endocrine function within scWAT or vWAT, or whether these changes reflect secondary responses to reduced adiposity and improved systemic metabolism.

Taken together, current evidence regarding the effects of dual GLP-1/GIP receptor agonists on mitochondrial metabolism and thermogenic remodeling of WAT remains inconclusive. Available animal studies suggest that tirzepatide may modulate mitochondrial markers and lipid metabolism within adipose tissue; however, evidence supporting induction of thermogenic remodeling remains limited. In humans, clinical evidence is currently restricted to circulating metabolic biomarkers. Therefore, further studies are needed to clarify whether the effects of this pharmacological strategy on WAT remodeling reflect local actions within adipose tissue or secondary adaptations associated with weight loss and improved metabolic health.

### Triple GLP-1/GIP/glucagon Receptor Agonists: Preliminary Effects on WAT Remodeling

Recently, triple GLP-1/GIP/glucagon receptor agonists, including retatrutide, have shown significant promise. Phase 2 clinical data reveal substantial reductions in body weight, exceeding 24% at a 12 mg dose over 48 weeks [113], consistent with potential synergistic metabolic effects of combined GLP-1R, GIPR, and GCGR agonism [114]. These clinical data establish retatrutide as a highly effective weight-loss therapy, but they do not directly define its effects on WAT remodeling. Evidence directly evaluating WAT remodeling remains scarce, and current mechanistic insights are derived primarily from animal model studies.

In an HFD-induced obesity mouse model, retatrutide treatment for 10 weeks improved metabolic parameters, including glucose homeostasis and dyslipidemia, while reducing both subcutaneous and visceral adiposity compared with untreated HFD controls. Transcriptomic analysis performed specifically in vWAT revealed that retatrutide downregulated inflammatory pathways, including chemokine, *Toll-like* receptor, TNF, and NF-κB signaling. In addition, retatrutide significantly upregulated key transcriptional regulators of fatty acid oxidation, *Ppara* and *Ppargc1a*, and restored the expression of genes involved in mitochondrial (*Cpt2*, *Acadvl*) and peroxisomal (*Acox1*, *Ehhadh*) β-oxidation, as well as genes critical for peroxisome biogenesis (*Pex3*) and mitochondrial membrane integrity (*Crls1*) [115].

However, these preclinical transcriptomic data are restricted to vWAT; corresponding scWAT analyses are not yet available. Because retatrutide also substantially reduces adiposity and improves systemic metabolism, future studies will be needed to determine whether these vWAT molecular adaptations reflect direct tissue-level remodeling or secondary responses to weight loss. Thus, retatrutide represents a promising emerging therapy, but evidence for depot-specific WAT remodeling not fully explained by weight loss remains preliminary.

**Fig. 1:**
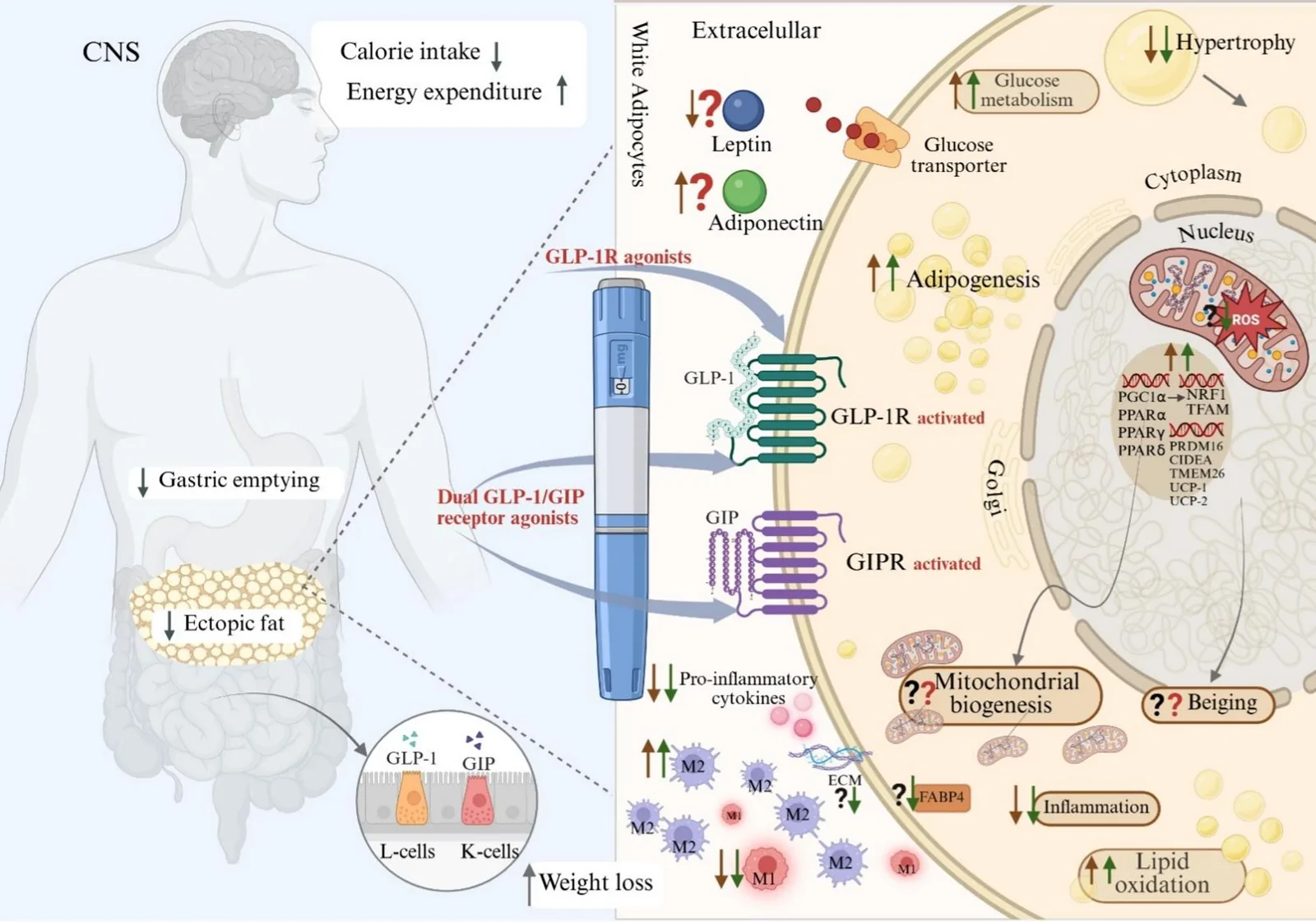
Incretin-based pharmacotherapies (GLP-1R agonists and dual GLP-1/GIP receptor agonists) are associated with improved obesity-related outcomes and may influence WAT remodeling through indirect mechanisms, including centrally mediated weight-loss–related effects, as well as through potential local actions within adipose tissue. Reported adipose effects span inflammation, mitochondrial/thermogenic programs, oxidative stress, and substrate handling. Brown arrows indicate findings from animal models; green arrows indicate findings from human studies. Question marks denote heterogeneous evidence and limited ability—especially in humans—to infer weight-independent (“beyond weight loss”) effects. Abbreviations: CIDEA: Cell Death Inducing DFFA Like Effector A, CNS: Central Nervous System, ECM: Extracellular Matrix, FABP4: Fatty Acid Binding Protein 4, GIP: Glucose-dependent Insulinotropic Polypeptide, GIPR: Glucose-dependent Insulinotropic Polypeptide Receptor, GLP-1: Glucagon-like Peptide-1, GLP-1R: Glucagon-like Peptide-1 Receptor, M1/M2: Macrophages Type 1 and Type 2, NRF1: Nuclear Respiratory Factor 1, PGC-1α: Peroxisome Proliferator-activated Receptor Gamma Coactivator 1-alpha, PPARα/γ/δ: Peroxisome Proliferator-activated Receptor alpha, gamma, and delta, PRDM16: PR Domain Containing 16, ROS: Reactive Oxygen Species, TFAM: Mitochondrial Transcription Factor A, TMEM26: Transmembrane Protein 26, UCP-1/2: Uncoupling Protein 1 and 2, WAT: White Adipose Tissue. Created with BioRender

Overall, incretin-based pharmacotherapies, including GLP-1R agonists, dual GLP-1/GIP receptor agonists, and emerging triple GLP-1/GIP/glucagon receptor agonists, are highly effective for weight loss and metabolic improvement in individuals with obesity. Emerging evidence suggests that these therapies may also influence WAT biology, including inflammatory, mitochondrial/thermogenic, and endocrine pathways (Table 1; Fig. 1). However, most tissue-level observations occur alongside reductions in adiposity and improvements in systemic metabolism, and human evidence remains limited. Thus, whether these molecular and tissue-level adaptations reflect direct WAT remodeling beyond weight loss remains unresolved and will require integrated in vitro, animal, and human adipose tissue studies.

**Table 1:** Summary of experimental studies on the effects of GLP-1 and dual GLP-1/GIP receptor agonists on WAT remodeling outcomes

Models	Therapies strategics	Experimental Groups	Protocol	WAT remodulation related outcomes	References
Animals studies					
Male 10-12-week-old C57BL/6 mice	Liraglutide	Control (vehicle-treated) vs. Liraglutide	Injected liraglutide at 100 µg/kg, twice daily for the last 17 days	Liraglutide activate the GLP-1R, thereby promoting pre-adipocyte proliferation and inhibition of apoptosis	Challa et al. 2012 [75]
Male 7-week-old KKAy mice (obese HFD-induced)	Liraglutide	Control (vehicle-treated) vs. Liraglutide	Injected liraglutide at 400 µg/kg twice daily for 12 weeks	Liraglutide treatment induced WAT browning: ↑ CIDEA, PPARγ, PRDM16 and UCP-1; ↑ mitochondrial marker genes such as PGC-1α and TFAM in inguinal and peripheral renal WAT	Zhu et al. 2016 [87]
Male 8-week-old C57BL/6 mice (HFD-induced obesity)	Liraglutide	Control (C) vs. Control + Liraglutide (C + LGT) vs. HFD vs. HFD + Liraglutide (HFD + LGT)	Liraglutide administrated subcutaneously at 200 µg/kg/day or saline solution (equivalent volume) for 5 weeks	Liraglutide resulted in ↑ UCP-1 expression, improved tissue architecture by reduced adipocyte size, ↑ vascularization in vWAT, and ↓ visceral fat mass	Touceda et al. 2024 [85]
Male C57BL/6 mice (obese HFD-induced)	Liraglutide	Control (C) vs. HFD vs. HFD + liraglutide	Liraglutide administrated subcutaneously at 0.1 mg/kg daily for 4 weeks	Liraglutide resulted in ↑ expression of UCP-1, PRDM16, and PPARα, improving mitochondrial function through the AMP-AMPK, SIRT-1 and PGC-1α signaling pathway	Zhou et al. 2019 [86]
Male 6-week-old C57BL/6 mice (HFD-induced)	Liraglutide	Control (C) vs. HFD vs. HFD + Liraglutide	Liraglutide administrated subcutaneously at 200 µg/kg/day for 20 weeks	Liraglutide resulted in ↓ body weight and fat mass. Improved adipose leptin resistance both scWAT and vWAT	Lyu et al. 2022 [94]
Male C57BL/6 mice (HFD-induced obesity)	Semaglutide	Control (C) vs. Control diet and semaglutide (C + S) vs. HFD vs. HFD and semaglutide (HFD + S)	Semaglutide administered subcutaneously to mice at 40 µg/kg once every 3 days (corresponding to 1.2 µg per animal for 4 weeks	Semaglutide resulted in ↓ fat mass (vWAT, -55%; scWAT, -40%); In vWAT: ↓ proinflammatory gene expressions: TNF-α, IL-6, IL-1β; ↓ macrophage infiltration; ↓Leptin. In scWAT: ↑ mitochondrial biogenesis and browning mediators: PGC-1α, NRF1, TFAM; PPARα, PPARγ, FNDC5, UCP-1, PRDM16 and the β3-adrenergic receptor.	Martins et al. 2022 [79]
Male 10-week-old C57BL/6J mice (HFD-induced obesity and weight regain models)	Tirzepatide	HFD-vehicle vs. LJ-4378 vs. Tirzepatide	Tirzepatide administered subcutaneously at 10 nmol/kg/day for 14 days	In vWAT: tirzepatide resulted in ↓ macrophage accumulation (F4/80) and CLS; ↓ Expression of pro-inflammatory markers such as TNF-α and Adgre1. In scWAT: ↑ Lipolysis and thermogenesis (↑ P-HSL, UCP-1 and COXIV)	Kim et al. 2025 [105]
Male C57BL/6J mice (HFD-induced obesity)	Tirzepatide	HFD-vehicle vs. HFD + Tirzepatide	Tirzepatide administered subcutaneously at a dose of 1.2 mg/kg twice a week for 12 weeks	Tirzepatide promoted apoptosis of macrophages by modulating the ERK signaling pathway in vWAT; ↓ TNF-α, IL-6, MCP-1, and IFN-γ in vWAT; ↓ both the size and weight of vWAT and scWAT	Xia et al. 2024 [24]
Male C57BL/6J mice (HFD-induced)	Tirzepatide + Mesoporous Polydopamine (MPDA)	HFD-vehicle vs. Tirzepatide vs. Tirzepatide + MPDA	Local inguinal WAT injection (TZP+MPDA) for 4 weeks	No significant changes in genes related to mitochondrial function or thermogenic activity; ↓ leptin resistance via activation of JAK2/STAT3 and AMPK/ACC1 pathways in scWAT	Mi et al. 2025 [108]
Humans studies					
Adults women with T2D and obesity	Liraglutide	Liraglutide vs. calorie restriction-based on dietetic counselling	Liraglutide started at a dose of 0.6 mg for 2 weeks and continued with 1.2 mg to week 16, once daily	Liraglutide resulted in a weight loss of 3.12 kg and ↓ visceral fat; ↑TNF-α MCP-1 and fibrosis/ECM remodeling markers in scWAT. No changes in eighter leptin and adiponectin scWAT expression	Pastel et al. 2017 [80]
Adults with T1D and overweight/obesity	Liraglutide	Placebo group vs. Liraglutide group	Liraglutide (1.8 mg/day) or placebo for 26 weeks	Liraglutide resulted in a weight loss of 3.38 kg. No significant differences in composition and function scWAT	Wegeberg et al. 2021 [82]
Overweight/obese adults with T1D	Liraglutide	Placebo group vs. Liraglutide group	Liraglutide (1.8 mg/daily) or placebo for 26 weeks	Liraglutide resulted in a weight loss of 3.9 kg; ↑ expression of ATGL and CPT-1 in scWAT	Ghanim et al. 2020 [81]
Adults with obesity	Semaglutide	Semaglutide group vs. placebo group	Semaglutide titrated up to maintenance dose (0.5 or 1.0 mg once weekly) for 24 weeks	Semaglutide resulted in weight loss (9%); modulates the pro-inflammatory profile on epicardial fat and ↓ levels of the adiposity marker FABP4	García-Vega et al. 2024 [83]

*ACC1* Acetyl-CoA Carboxylase 1, *Adgre1* Adhesion G Protein-Coupled Receptor E1, *AMPK* AMP-activated Protein Kinase, *ATGL* Adipose Triglyceride Lipase, *CIDEA* Cell Death Inducing DFFA Like Effector A, *CLS* Crown-Like Structures, *COXIV* Cytochrome c Oxidase Subunit 4, *CPT-1* Carnitine Palmitoyltransferase 1, *ECM* Extracellular Matrix, *ERK* Extracellular Signal-Regulated Kinase, *FABP4* Fatty Acid Binding Protein 4, *FNDC5* Fibronectin Type III Domain Containing 5, *GIP* Glucose-dependent Insulinotropic Polypeptide, *GIPR* Glucose-dependent Insulinotropic Polypeptide Receptor, *GLP-1* Glucagon-like Peptide-1, *GLP-1R* Glucagon-like Peptide-1 Receptor, *HFD* High-Fat Diet, *IFN-γ* Interferon gamma, *IL-1β/6* Interleukin 1 beta and 6, *JAK2* Janus Kinase 2, *LepR* Leptin Receptor, *M1/M2* Macrophages Type 1 and Type 2, *MCP-1* Monocyte Chemoattractant Protein-1, *MDA* Malondialdehyde, *NRF1* Nuclear Respiratory Factor 1, *P-CREB* Phosphorylated cAMP Response Element-Binding Protein, *PGC-1α* Peroxisome Proliferator-activated Receptor Gamma Coactivator 1-alpha, *P-HSL* Phosphorylated Hormone-Sensitive Lipase, *PPARα/γ* Peroxisome Proliferator-activated Receptor alpha and gamma, *PRDM16* PR Domain Containing 16, *scWAT* Subcutaneous White Adipose Tissue, *SERCA* Sarco/Endoplasmic Reticulum Calcium-ATPase, *SIRT-1* Sirtuin 1, *STAT3* Signal Transducer and Activator of Transcription 3, *T1D/T2D* Type 1 and Type 2 Diabetes, *TFAM* Mitochondrial Transcription Factor A, *TNF-α* Tumor Necrosis Factor alpha, *UCP-1* Uncoupling Protein 1, *vWAT* Visceral White Adipose Tissue, *WAT* White Adipose Tissue

Sex is another important source of heterogeneity that remains insufficiently addressed in this literature. WAT is highly sexually dimorphic, with sex differences in depot distribution, adipocyte size, inflammatory tone, mitochondrial and thermogenic programs, and adipokine secretion [116]. However, most studies evaluating the effects of GLP-1R agonists, dual GLP-1/GIP receptor agonists, or triple GLP-1/GIP/glucagon receptor agonists on WAT have not reported adipose outcomes stratified by sex, and several preclinical studies have relied primarily on male rodents. Thus, whether incretin-based therapies induce distinct remodeling responses in scWAT versus vWAT in females and males remains largely unresolved [116, 117].

## Bariatric Surgery and Remodeling of WAT

### Bariatric Surgery Procedures

Bariatric surgery procedures include sleeve gastrectomy (SG), which reduces stomach volume, and Roux-en-Y gastric bypass (RYGB), which combines gastric restriction with intestinal bypass [118]. These procedures can be performed laparoscopically and account for more than 90% of currently used bariatric techniques [3, 119]. SG involves longitudinal stapling and removal of approximately 85% of the stomach, resulting in a long, narrow gastric pouch that reduces gastric volume while preserving the pylorus [3, 119, 120]. RYGB involves creation of a small gastric pouch that is directly anastomosed to the jejunum, forming the alimentary limb. Digestive fluids, including bile and pancreatic secretions, encounter ingested nutrients only where the alimentary limb joins the biliopancreatic limb, forming the common channel, which is the primary site of nutrient digestion and absorption [120].

Importantly, bariatric surgery should not be viewed solely as an intervention that induces substantial weight loss. The gastrointestinal anatomical changes resulting from surgery, together with subsequent physiological adaptations, may contribute to adipose tissue changes not fully explained by reduced adiposity through effects on inflammatory, mitochondrial, thermogenic, and endocrine pathways [121].

Thus, surgery-specific biology, including altered nutrient delivery, rapid enteroendocrine responses, bile acid signaling, microbiome remodeling, and gut–brain communication, may contribute to systemic metabolic improvement and potentially to adipose tissue adaptation, although the extent to which these pathways act independently of weight loss remains incompletely defined. We therefore summarize evidence for postoperative WAT changes across inflammatory, mitochondrial/thermogenic, endocrine, and incretin-related domains, while highlighting where concurrent weight loss limits mechanistic interpretation.

## Effects of Bariatric Surgery on WAT Remodeling: Modulation of Inflammatory, Mitochondrial, Thermogenic, and Endocrine Pathways

### Modulation of WAT Inflammation Following Bariatric Surgery

Bariatric surgery is associated with favorable WAT remodeling, including reductions in adipocyte hypertrophy and inflammatory signaling, although the extent to which these effects are surgery-specific remains difficult to determine [122–124]. In animal models, RYGB is associated with reduced adipocyte size, attenuated inflammatory cell infiltration, including CD3 + T lymphocytes, and decreased expression of pro-inflammatory cytokines, including IFN-γ, MCP-1, TNFα, macrophage inflammatory protein-1-alpha (MIP-1α), and MIP-1β, in vWAT [123].

Human studies broadly support postoperative improvements in WAT morphology and inflammatory tone, although most tissue-level evidence comes from scWAT. In clinical studies of RYGB, subcutaneous adipocyte size and area progressively decrease during the first postoperative year [124, 125]. This morphological shift is further characterized by ECM remodeling within scWAT [126]. Notably, reductions in adipocyte area and volume appear to be sustained beyond the second year after surgery [127, 128].

Global transcriptomic and methylomic analyses further indicate molecular remodeling of scWAT after bariatric surgery in individuals with severe obesity. This remodeling is primarily characterized by gene downregulation and hypermethylation of loci associated with immune and inflammatory signaling [129]. For instance, a clinical trial demonstrated a significant decrease in scWAT macrophage counts, specifically mature HAM56 + cells, three months after RYGB [122]. These results are corroborated by studies showing a significant reduction in M1-type CD40 + cells alongside an increase in M2-type CD206 + and CD163 + cells in scWAT during the same postoperative period [130]. Among pro-inflammatory mediators, MCP-1 levels in scWAT appear to decline significantly within three months [122]. Conversely, other evidence indicates that expression of IL-6, IL-18, and TNF-α may remain unchanged 24 weeks after RYGB, even when significant weight loss is accompanied by improved glycemic control and insulin sensitivity [125]. However, because most human studies assess inflammatory markers before and after surgery without weight-matched dietary or pharmacological comparators, it remains difficult to determine whether these inflammatory changes reflect surgery-specific mechanisms or secondary consequences of reduced adiposity.

### Mitochondrial Remodeling and Potential Thermogenic Modulation of WAT Following Bariatric Surgery

Beyond structural, immunological, and inflammatory remodeling, emerging evidence suggests that bariatric surgery may influence mitochondrial pathways and thermogenic-related markers in WAT. In animal models of obesity, RYGB has been associated with upregulation of PGC-1α, NRF1, UCP-1, UCP-2, and UCP-3 in WAT [131].

In humans, a retrospective study comparing diet-induced and bariatric surgery-induced weight loss analyzed mitochondrial-related RNA expression profiles in scWAT. Transcriptomic and proteomic analyses showed that diet-induced weight loss was accompanied by downregulation of OXPHOS-related transcripts, whereas RYGB-induced weight loss was associated with upregulation of OXPHOS pathways 12 months after surgery. When mitochondrial-related gene expression was analyzed according to the percentage of weight loss, OXPHOS transcripts remained downregulated after diet-induced weight loss but upregulated after surgery-induced weight loss [132], suggesting that mitochondrial remodeling in scWAT may vary by type of weight-loss intervention and is therefore relevant to the “beyond weight loss” framework. Consistent with these findings, ex vivo assessments of scWAT and vWAT explants showed improved mitochondrial oxidative capacity and upregulation of OXPHOS-related pathways in both adipose depots one year after RYGB [133].

### Endocrine Modulation of WAT Following Bariatric Surgery

Compared with inflammatory and mitochondrial outcomes, tissue-level evidence for endocrine remodeling after bariatric surgery is more limited and is largely restricted to scWAT or circulating biomarkers. In rodents, SG has been shown to reduce body weight and fat mass without inducing immediate changes in central leptin sensitivity [134].

In humans, leptin expression in scWAT is reduced four weeks after surgery, while adiponectin levels increase in the same depot at 4 and 24 weeks following weight loss, suggesting improved adipose tissue endocrine function alongside reduced adipocyte size over the same period [125]. Furthermore, *LepR* expression in humans appears to increase as subcutaneous adipocyte size decreases eight months after RYGB [124].

Regarding RBP4, human clinical trials have reported significant decreases in circulating levels 6 and 10 months post-surgery, which correlate with improved insulin sensitivity and weight reduction [135–137]. Although these findings suggest that bariatric surgery may be associated with favorable endocrine changes in scWAT, evidence on vWAT remains scarce, and most available evidence is derived from circulating biomarkers rather than analyses at the tissue level [135–139].

In this context, although the available evidence remains limited, bariatric surgery appears to be associated with coordinated WAT changes, including attenuation of inflammatory pathways, improved mitochondrial oxidative metabolism, modulation of adipose tissue endocrine function, reductions in adipocyte hypertrophy, extracellular matrix remodeling, and improvements in metabolic parameters following surgery. However, as summarized in Table 2, most studies compare baseline with postoperative measurements or include control groups without an equivalent degree of weight loss, limiting determination of whether these adaptations are surgery-specific or secondary to reduced adiposity and improved systemic metabolism. Notably, one of the few studies directly comparing diet-induced and surgery-induced weight loss reported divergent effects on mitochondrial-related gene expression in scWAT [132], suggesting that the type of weight-loss intervention may influence mitochondrial remodeling. Nevertheless, direct evidence demonstrating surgery-specific WAT remodeling not fully explained by weight loss remains scarce, and distinguishing surgery-specific effects from weight loss-related physiological adaptations remains an important challenge for the field.

**Table 2:** Summary of experimental studies on the effects of bariatric surgery on WAT remodeling outcomes

Models	Therapies strategics	Experimental Groups	WAT remodulation related outcomes	References
Animals studies				
Male C57BL/6 J mice (HFD-induced obesity)	RYGB	Sham surgery vs. RYGB vs. Caloric restriction (CR)	At 4 weeks postsurgery: ↓ body weight and fat mass; scWAT browning via upregulation of PGC1α, NRF1, UCP1, UCP2 and UCP3	Huang et al. 2022 [131]
Male 12-week-old db/db mice (Type 2 Diabetic model)	RYGB	Sham surgery vs. RYGB	At 8 weeks postsurgery: ↓ adipocyte size; ↓ inflammatory cell infiltration CD3 + T-lymphocytes); ↓ pro-inflammatory cytokine (IFN-γ, MCP-1, TNFα); ↓ macrophage (MIP-1α and MIP-1β) in vWAT	Zhang et al. 2011 [123]
**Humans studies**				
Women with morbidly obesity (BMI 48.0 ± 6.8 kg/m²)	RYGB	Baseline vs. 3 months postsurgery vs. Control group	At 3 months postsurgery: ↓ body weight (Δ -22.1 kg); ↓ macrophage infiltration (marked by mature HAM56 + cells) in scWAT	Cancello et al. 2005 [122]
Women with obesity (BMI 43.8 ± 3.4 kg/m^2^)	RYGB	Baseline vs. 3 months postsurgery vs. Control group	At 3 months postsurgery: ↓ body weigh by 18.9 kg; ↓ M1-type (CD40 + cells); ↑ M2-type (CD206 + and CD163 + cells) in scWAT	Aron-Wisnewsky et al. 2009 [130]
Adults with obesity (BMI 36.8 ± 4 kg/m^2^) and T2D	RYGB	Baseline vs. 4 weeks and 24 weeks postsurgery vs. Control group	In scWAT: ↓ adipocyte size more at 4 than at 24 weeks; at 4 weeks: ↓ glucose uptake per adipocyte; ↓leptin expression; no significant changes in the expression of inflammation-related genes (IL-6, IL-18, and TNF-α); ↑ adiponectin levels at both 4 and 24 weeks	Katsogiannos et al. 2019 [125]
Adults with seven obesity (BMI 49.3 ± 2.5 kg/m²)	RYGB	Baseline vs. 8 months postsurgery	At 8 months postsurgery: Lost ∼ 36% of initial body weight; ↓ size and area of scWAT; ↑ LepR expression	Tamez et al. 2017 [124]
Women with morbidly obesity (BMI 48.0 ± 6.8 kg/m²)	RYGB or SG or Gastric Banding	Baseline vs. 1-year postsurgery	At 1-year postsurgery: ↓ expression of genes encoding ECM components (↓COL3A1, COL6A1, COL6A2, ELN) in scWAT	Liu et al. 2016 [126]
Adults with overweight or obesity	RYGB	Baseline vs. 1-year postsurgery vs. Diet-induced weight loss (LCD)	At 1-year postsurgery: ↓ body weight by 22.4%; upregulation of OXPHOS pathways in scWAT	van der Kolk et al. 2021 [132]
Adults with obesity	RYGB or SG	Baseline vs. 1-year postsurgery vs. Control group	At 1-year postsurgery: ↑ mitochondrial oxidative capacity mediated via upregulation of OXPHOS in scWAT and vWAT explants	Pinho et al. 2025 [133]
Women with obesity	RYGB	Baseline vs. 2-year postsurgery	At 2-year postsurgery: ↓ body weight by 33%, accompanied by ↓ subcutaneous fat cell volume	Andersson et al. 2014 [127]

*BMI* Body Mass Index, *CD3+/40+/163+/206+* Cluster of Differentiation 3, 40, 163, and 206, *COL3A1/6A1/6A2* Collagen Type III Alpha 1 Chain, and Type VI Alpha 1 and 2 Chains, *CR* Caloric Restriction, *ECM* Extracellular Matrix, *ELN* Elastin, *HAM56* Human Alveolar Macrophage 56, *HFD* High-Fat Diet, *IFN-γ* Interferon gamma, *IL-6/18* Interleukin 6 and 18, *LCD* Low-Calorie Diet, *LepR* Leptin Receptor, *M1/M2* Macrophages Type 1 and Type 2, *MCP-1* Monocyte Chemoattractant Protein-1, *MIP-1α/1β* Macrophage Inflammatory Protein 1-alpha and 1-beta, *NRF1* Nuclear Respiratory Factor 1, *OXPHOS* Oxidative Phosphorylation, *PGC1α* Peroxisome Proliferator-activated Receptor Gamma Coactivator 1-alpha, *RYGB* Roux-en-Y Gastric Bypass, *scWAT* Subcutaneous White Adipose Tissue, *SG* Sleeve Gastrectomy, *T2D* Type 2 Diabetes, *TNF-α* Tumor Necrosis Factor alpha, *UCP1/2/3* Uncoupling Protein 1, 2, and 3, *vWAT* Visceral White Adipose Tissue, *WAT* White Adipose Tissue

Sex is another important source of heterogeneity that may shape postoperative WAT remodeling. Many clinical bariatric cohorts include a high proportion of females, yet tissue-level studies rarely test sex-specific responses in adipocyte morphology, inflammation, ECM remodeling, mitochondrial oxidative capacity, thermogenic markers, or endocrine signaling [140, 141]. Given established sex differences in fat distribution and WAT biology [116], future paired-depot studies should evaluate whether scWAT and vWAT remodeling after SG or RYGB differs between females and males and whether these differences contribute to variability in metabolic outcomes.

Because bariatric surgery also alters gut hormone dynamics, including postprandial GLP-1 and GIP responses, changes in incretin physiology may provide one mechanistic link between gastrointestinal remodeling, improved glycemic control, altered appetite regulation, and potentially WAT function.

#### Impacts of Bariatric Surgery on Incretin Physiology

Many beneficial metabolic outcomes of bariatric surgery are attributed to postoperative shifts in gut hormone dynamics, including changes in GLP-1 and GIP [120]. Circulating GLP-1 and GIP levels measured postprandially or in response to an oral glucose load increase significantly as early as one month after intervention, a physiological shift that persists for up to three years [142, 143]. Clinical evidence has demonstrated that RYGB promotes a rapid increase in the magnitude of postprandial GLP-1 within the first month, which remains elevated for at least two years after surgery. Despite temporal variation in these levels, this enhanced incretin response is closely associated with improved glucose tolerance and insulin sensitivity [144].

The mechanisms underlying this sustained post-surgical increase in GLP-1 remain a subject of investigation but are largely attributed to accelerated nutrient delivery to the distal small intestine, a region with a high density of enteroendocrine L-cells, whose stimulation serves as a primary trigger for GLP-1 release following both SG and RYGB in animal models and humans [142–145]. Furthermore, RYGB accelerates gastric emptying and intestinal transit, thereby potentiating the postprandial GLP-1 excursion [144, 146]. Intestinal mucosal adaptations and alterations in the gut microbiota composition may also contribute to the long-term elevation of GLP-1 levels [147, 148].

Unlike GLP-1, circulating GIP levels, which reflect secretion by K cells in the upper gastrointestinal tract, show greater variability across studies. This inconsistency may be influenced by surgical technique, time elapsed since the procedure, and pathological conditions such as T2D [5, 57, 143]. Several clinical trials have reported reduced postprandial GIP secretion between 1 and 4 weeks following RYGB [145, 149]. These findings suggest that surgical anastomosis limits nutrient exposure to the duodenum and proximal jejunum, regions with the highest K cell concentration, thereby reducing GIP stimulation [145, 149–151].

Recent evidence suggests that incretin profiles after bariatric surgery may correlate with long-term weight trajectories. A study examining patients ten years after RYGB found that postprandial glucose and GIP levels were positively correlated with weight regain. Conversely, postprandial GLP-1 and glucagon levels were negatively correlated with weight regain, suggesting that weight regain may either impair incretin signaling or reflect weakened GLP-1-mediated satiety that contributes to increased caloric intake and subsequent weight gain, potentially exacerbated by elevated GIP levels [152].

**Fig. 2:**
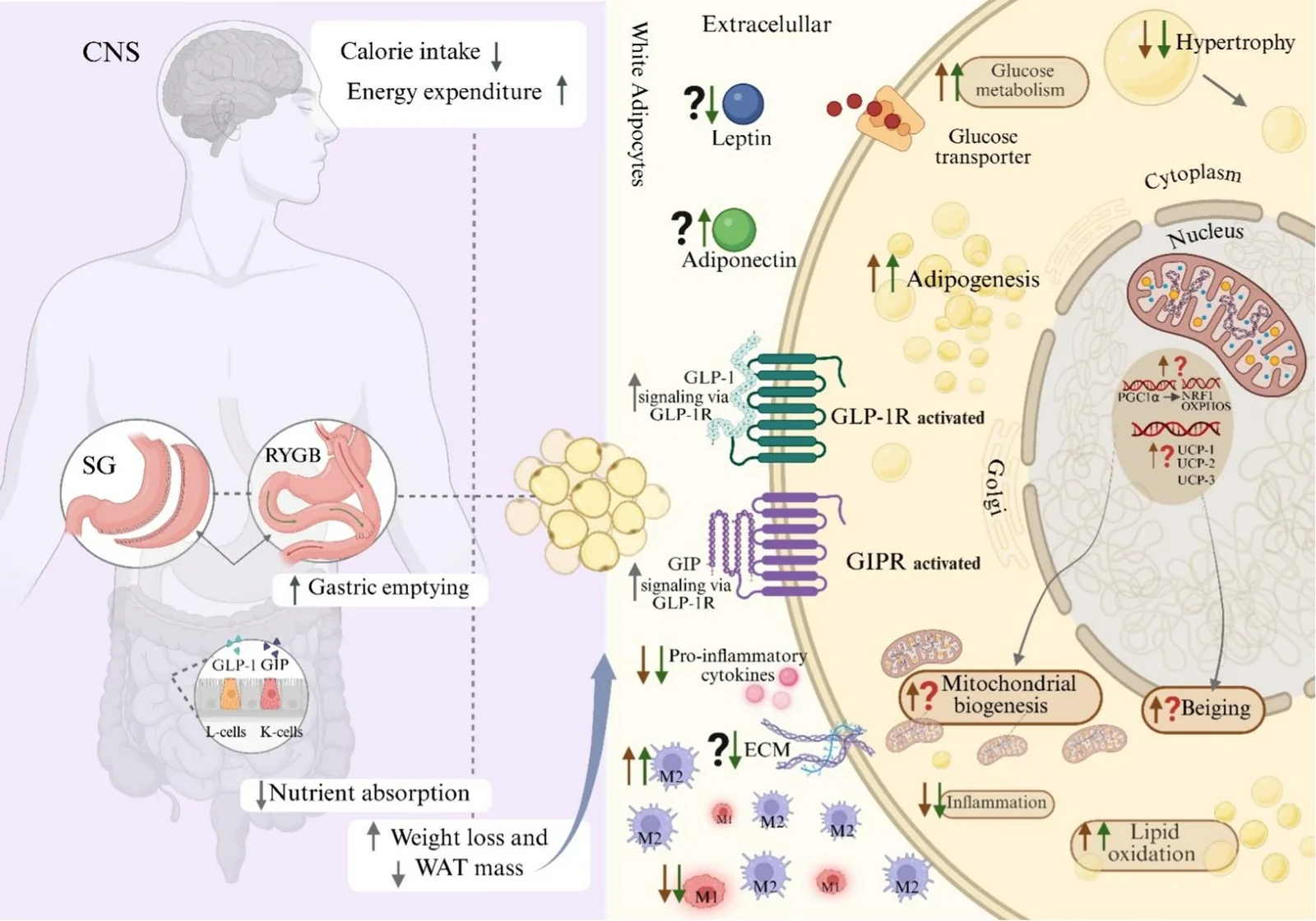
Bariatric surgery (e.g., RYGB and SG) is associated with substantial weight loss and improved metabolic outcomes and may influence WAT remodeling through reduced adiposity/negative energy balance and surgery-associated endocrine changes (including altered incretin physiology). Reported adipose effects span inflammation, mitochondrial/thermogenic programs, and substrate handling. Brown arrows indicate findings from animal models; green arrows indicate findings from human studies. Question marks denote heterogeneous evidence and limited ability in humans to disentangle weight-dependent effects from potential weight-independent mechanisms. Abbreviations: ECM: Extracellular Matrix, GIP: Glucose-dependent Insulinotropic Polypeptide, GIPR: Glucose-dependent Insulinotropic Polypeptide Receptor, GLP-1: Glucagon-like Peptide-1, GLP-1R: Glucagon-like Peptide-1 Receptor, M1/M2: Macrophages Type 1 and Type 2, NRF1: Nuclear Respiratory Factor 1, PGC-1α: Peroxisome Proliferator-activated Receptor Gamma Coactivator 1-alpha, RYGB: Roux-en-Y Gastric Bypass, SG: Sleeve Gastrectomy, UCP-1/2/3: Uncoupling Proteins 1, 2, and 3, WAT: White Adipose Tissue. Created with BioRender

Ultimately, GLP-1 and GIP are important physiological mediators of several metabolic effects of bariatric surgery. Surgery-induced stimulation of these hormones, primarily at the intestinal level, contributes to endocrine and metabolic responses involved in glycemic control and weight loss [120]. Nevertheless, further research is required to elucidate the complex dynamics of incretin alterations following metabolic interventions. Figure 2 illustrates physiological and metabolic impacts of bariatric surgery on WAT remodeling.

### Magnitude of Weight Loss, Long-Term Management, and Health Outcomes: Incretin-Based Therapies and Bariatric Surgery

Despite scientific advances and increasing demand for both incretin-based pharmacotherapy and bariatric procedures, the magnitude of weight reduction and long-term weight maintenance remain subjects of intense debate [153, 154]. A systematic review and meta-analysis comparing weight loss between GLP-1R agonists and bariatric surgery demonstrated that, while surgery still confers superior weight reduction, it achieves similar improvements in glycemic control compared with various GLP-1R agonists [153]. More recent network meta-analyses further indicate that, compared with GLP-1R agonists such as liraglutide and semaglutide, bariatric surgery achieves significantly greater reductions in body weight (-11.7 kg), BMI (-4.5 kg/m²), waist circumference (-12.6 cm), and HbA1c (-0.5%) at < 104 weeks, with differences in percent total weight loss and body weight (-14.6 kg) remaining significant at ≥ 104 weeks. However, in subgroup analyses restricted to tirzepatide, differences compared with bariatric surgery were not statistically significant, suggesting that this dual agonist may approach surgical efficacy [155].

Critically, the comparative effectiveness of pharmacotherapy versus surgery in reducing mortality and major adverse cardiovascular events (MACEs) remains a subject of active research. A cohort study of 6070 patients compared all-cause mortality and non-fatal MACEs between bariatric surgery and GLP-1R agonists. Among patients with obesity and a diabetes duration of ≤ 10 years (without prior ischemic heart disease or congestive heart failure), bariatric surgery was associated with a 62% reduction in mortality compared to GLP-1R agonists. No significant mortality differences were observed in those with a longer duration of diabetes, nor were there differences in MACE risk across the total cohort [156]. Conversely, other data reported a lower risk for MACEs among diabetic patients who underwent metabolic surgery compared to those treated with GLP-1R agonists, though these findings are limited by a lack of differentiation regarding prior CVD history [157]. Such heterogeneity underscores the need for further studies to confirm these associations and elucidate the underlying mechanisms.

Another decisive factor is the management of long-term weight maintenance. A recent systematic review and meta-analysis evaluated the rate of weight regain after the cessation of weight management medications in adults with overweight or obesity. The findings demonstrated a weight regain of approximately 0.8 kg per month, with a projected return to baseline weight within 1.5 years following the discontinuation of semaglutide or tirzepatide treatment [154]. However, it is crucial to emphasize that, as with any pharmacological intervention, clinical benefits may be lost upon cessation, leading to weight recidivism. Interestingly, another systematic review compared current evidence on weight regain following weight loss achieved through lifestyle intervention, semaglutide, and bariatric surgery in adults with obesity. Bariatric surgery achieved sustainable results, with a total weight loss of > 10% maintained for over 5 years. In contrast, lifestyle-based strategies provided moderate results, largely depending on consistency and follow-up, while semaglutide offered potent short-term efficacy but was associated with weight recurrence after treatment interruption [158].

Beyond promoting weight loss, incretin-based therapies may also modulate the gut microbiome. In animal models of HFD-induced obesity, subcutaneous semaglutide for 18 days and tirzepatide for 14 days were associated with changes in gut microbiota composition and microbial dysbiosis. These alterations were accompanied by inverse associations between the relative abundance of selected bacterial taxa and weight gain [159, 160]. However, it remains unclear whether these microbiota alterations represent direct effects of incretin-based therapies or occur secondary to weight loss and accompanying metabolic improvements. Consequently, whether microbiota-mediated changes directly contribute to WAT remodeling remains to be established. In contrast, available evidence suggests a role for the gut microbiota in regulating adipose tissue metabolism following bariatric surgery. Using paired fecal microbiota transplantation (FMT) from patients before and after RYGB into germ-free mice, post-surgical microbiota improved insulin sensitivity and was associated with reduced expression of inflammatory genes, including *Tnf-α*, *Ccl2* (MCP-1), and *Elane* (neutrophil elastase), in vWAT, as well as reduced *Tnf-α* and *Elane* expression in scWAT. These findings suggest that gut microbiota alterations may contribute to improvements in adipose tissue inflammation and metabolic health following RYGB [161]. Thus, compared with incretin-based therapies, bariatric surgery currently has stronger experimental support for microbiota-mediated gut–adipose signaling, although whether these pathways causally explain distinct adipose tissue remodeling outcomes remains unresolved.

## Combined Effects of Incretin-Based Therapies After Bariatric Surgery

The role of incretin-based therapies as adjunctive treatments for patients with insufficient weight loss or weight recurrence after bariatric surgery has also emerged as a critical area of investigation [162]. Specifically, semaglutide (1 mg/week) showed superior performance in achieving weight loss (12.92%) compared to liraglutide (3.0 mg/day; 8.77%) over 12 months in patients with post-surgical weight regain [163]. Furthermore, tirzepatide demonstrated even greater weight reduction compared to semaglutide over six months in individuals more than five years post-surgery [164]. Beyond weight management, 12-month adjunctive therapy with both semaglutide and liraglutide has been shown to significantly reduce HbA1c levels, improve lipid profiles, and lower blood pressure in post-bariatric individuals [165–170].

While bariatric surgery remains associated with superior outcomes in terms of durable weight loss and comorbidity prevention [171], both interventions are powerful tools in the treatment of obesity. Importantly, these strategies should be viewed as complementary rather than mutually exclusive, given that obesity is a chronic disease requiring constant management. Within this framework, lifestyle modifications, including physical exercise and dietary interventions, remain essential tools to investigate the hierarchy, interaction, and reversibility of obesity-related pathologies across the lifespan.

## Future Directions

This review synthesizes molecular mechanisms underlying pathological WAT remodeling in obesity, particularly in scWAT and vWAT, emphasizing that both incretin-based therapies and bariatric procedures are associated with beneficial adipose tissue changes. Selected evidence suggests that some effects are not fully explained by the magnitude of weight loss alone and may contribute to improved metabolic outcomes and remission of comorbidities such as T2D. However, several critical questions remain. Future studies should investigate how tissue remodeling across structural, morphological, inflammatory, mitochondrial, thermogenic, and endocrine dimensions interacts with age and whether these improvements are preserved long term. Additionally, spatial mapping of adipose tissue to reveal cell types, tissue architecture, and remodeling patterns provides a critical perspective on the intrinsic heterogeneity of WAT [172].

The efficacy of these interventions is modulated by multiple factors, including sex, genetics, pharmacological dosage, surgical technique, and baseline metabolic status. Such heterogeneity requires future studies to prioritize longitudinal designs with rigorous variable control to mitigate confounding. Age-stratified models integrated with translational research will be essential to ensure reliable and reproducible results.

More recently, pharmacological combinations aimed at preserving lean mass during obesity treatment have been investigated. Bimagrumab is an investigational antibody that binds to activin type II receptors (ActRIIA and ActRIIB), thereby blocking myostatin signaling and favoring preservation of muscle mass. Furthermore, bimagrumab binds to receptors on the adipocyte membrane, deactivating the activin-ALK7-Smad axis and increasing lipid mobilization and lipolysis. Phase 2 clinical data demonstrate that the combination of bimagrumab and semaglutide promoted greater weight loss and a significantly lower reduction in lean mass compared with semaglutide alone over 48 weeks, accompanied by reductions in visceral fat percentage and waist circumference [173].

Although weight reduction is consistently achieved by both incretin-based therapies and bariatric surgery, the precise underlying mechanisms are not yet fully elucidated, and long-term outcomes remain uncertain. This uncertainty calls for experimental designs that can translate mechanistic findings into robust, clinically relevant evidence.

Finally, the integration of non-pharmacological strategies, such as physical exercise and dietary interventions, remains widely recommended to enhance adipose tissue plasticity and remodeling. As a limitation, it should be noted that this work is a narrative review and does not follow a systematic protocol for study selection, which may introduce selection bias. Nevertheless, this review offers an updated synthesis of current evidence, identifies critical knowledge gaps, and proposes directions for future investigations aimed at establishing WAT as a central target in obesity treatment, notwithstanding the inherent complexities of adipose tissue collection, particularly in human clinical settings.

## Conclusion

In obesity, adipose tissue undergoes pathological remodeling characterized by structural, morphological, and functional alterations that contribute to metabolic comorbidities. Despite significant scientific progress, harnessing the therapeutic potential of interventions aimed at beneficial adipose tissue remodeling remains a critical challenge. In recent years, pharmacological modalities, including incretin-based therapies, and surgical interventions, such as bariatric surgery, have advanced substantially. While bariatric surgery appears to yield superior outcomes regarding durable weight loss, both interventions are associated with changes in cellular and molecular pathways relevant to WAT remodeling, although the degree to which these changes are not fully explained by weight loss remains incompletely defined. Emerging pharmacotherapies may further narrow the efficacy gap between pharmacological and surgical outcomes. Ultimately, integration of non-pharmacological strategies, including exercise and dietary interventions, is indispensable for promoting synergistic tissue-level and metabolic effects in the global fight against obesity.

## Data Availability

No datasets were generated or analysed during the current study.

## References

[1] World Health Organization. WHO acceleration plan to stop obesity. Updated July 3. 2023. Accessed May 5, 2025. https://www.who.int/publications/i/item/9789240075634

[2] Safaei M, Sundararajan EA, Driss M, Boulila W, Shapi’i A. A systematic literature review on obesity: understanding the causes and consequences of obesity and reviewing various machine learning approaches used to predict obesity. Comput Biol Med. 2021;136:104754. 10.1016/j.compbiomed.2021.104754. 34426171

[3] Elmaleh-Sachs A, Schwartz JL, Bramante CT, Nicklas JM, Gudzune KA, Jay M. Obesity management in adults: a review. JAMA. 2023;330(20):2000–15. 10.1001/jama.2023.19897. 38015216 PMC11325826

[4] Maniyadath B, Zhang Q, Gupta RK, Mandrup S. Adipose tissue at single-cell resolution. Cell Metab. 2023;35(3):386–413. 10.1016/j.cmet.2023.02.002. 36889280 PMC10027403

[5] Samms RJ, Coghlan MP, Sloop KW. How may GIP enhance the therapeutic efficacy of GLP-1? Trends Endocrinol Metab. 2020;31(6):410–21. 10.1016/j.tem.2020.02.006. 32396843

[6] Blüher M. Understanding adipose tissue dysfunction. J Obes Metab Syndr. 2024;33(4):275–88. 10.7570/jomes24013. 39734091 PMC11704217

[7] Rosen ED, Spiegelman BM. What we talk about when we talk about fat. Cell. 2014;156(1–2):20–44. 10.1016/j.cell.2013.12.012. 24439368 PMC3934003

[8] Chait A, den Hartigh LJ. Adipose tissue distribution, inflammation and its metabolic consequences, including diabetes and cardiovascular disease. Front Cardiovasc Med. 2020;7:22. 10.3389/fcvm.2020.00022. 32158768 PMC7052117

[9] Cannon B, Nedergaard J. Brown adipose tissue: function and physiological significance. Physiol Rev. 2004;84(1):277–359. 10.1152/physrev.00015.2003. 14715917

[10] Sacks H, Symonds ME. Anatomical locations of human brown adipose tissue: functional relevance and implications in obesity and type 2 diabetes. Diabetes. 2013;62(6):1783–90. 10.2337/db12-1430. 23704519 PMC3661606

[11] Cheng L, Wang J, Dai H, Duan Y, An Y, Shi L, et al. Brown and beige adipose tissue: a novel therapeutic strategy for obesity and type 2 diabetes mellitus. Adipocyte. 2021;10(1):48–65. 10.1080/21623945.2020.1870060. 33403891 PMC7801117

[12] Auger C, Kajimura S. Adipose tissue remodeling in pathophysiology. Annu Rev Pathol. 2023;18:71–93. 10.1146/annurev-pathol-042220-023633. 36070562 PMC9877135

[13] Reilly SM, Saltiel AR. Adapting to obesity with adipose tissue inflammation. Nat Rev Endocrinol. 2017;13(11):633–43. 10.1038/nrendo.2017.90. 28799554

[14] Gao F, Litchfield B, Wu H. Adipose tissue lymphocytes and obesity. J Cardiovasc Aging. 2024;4:5. 10.20517/jca.2023.38. 38455510 PMC10919906

[15] Montes VN, Turner MS, Subramanian S, Ding Y, Hayden-Ledbetter M, Slater S, et al. T cell activation inhibitors reduce CD8 + T cell and pro-inflammatory macrophage accumulation in adipose tissue of obese mice. PLoS ONE. 2013;8(7):e67709. 10.1371/journal.pone.0067709. 23844072 PMC3699637

[16] Travers RL, Motta AC, Betts JA, Bouloumié A, Thompson D. The impact of adiposity on adipose tissue-resident lymphocyte activation in humans. Int J Obes (Lond). 2015;39(5):762–9. 10.1038/ijo.2014.195. 25388403 PMC4424387

[17] Williams AS, Kang L, Wasserman DH. The extracellular matrix and insulin resistance. Trends Endocrinol Metab. 2015;26(7):357–66. 10.1016/j.tem.2015.05.006. 26059707 PMC4490038

[18] Sun K, Kusminski CM, Scherer PE. Adipose tissue remodeling and obesity. J Clin Invest. 2011;121(6):2094–101. 10.1172/JCI45887. 21633177 PMC3104761

[19] Menghini R, Menini S, Amoruso R, Fiorentino L, Casagrande V, Marzano V, et al. Tissue inhibitor of metalloproteinase 3 deficiency causes hepatic steatosis and adipose tissue inflammation in mice. Gastroenterology. 2009;136(2):663–e6724. 10.1053/j.gastro.2008.10.079. 19027012

[20] De Pauw A, Tejerina S, Raes M, Keijer J, Arnould T. Mitochondrial (dys)function in adipocyte (de)differentiation and systemic metabolic alterations. Am J Pathol. 2009;175(3):927–39. 10.2353/ajpath.2009.081155. 19700756 PMC2731113

[21] Heinonen S, Buzkova J, Muniandy M, Kaksonen R, Ollikainen M, Ismail K, et al. Impaired mitochondrial biogenesis in adipose tissue in acquired obesity. Diabetes. 2015;64(9):3135–45. 10.2337/db14-1937. 25972572

[22] Chattopadhyay M, Khemka VK, Chatterjee G, et al. Enhanced ROS production and oxidative damage in subcutaneous white adipose tissue mitochondria in obese and type 2 diabetes subjects. Mol Cell Biochem. 2015;399:95–103. 10.1007/s11010-014-2236-7. 25312902

[23] Heinonen S, Jokinen R, Rissanen A, Pietiläinen KH. White adipose tissue mitochondrial metabolism in health and in obesity. Obes Rev. 2020;21(2):e12958. 10.1111/obr.12958. 31777187

[24] Xia Y, Jin J, Sun Y, Kong X, Shen Z, Yan R, Huang R, Liu X, Xia W, Ma J, Zhu X, Li Q, Ma J. Tirzepatide’s role in targeting adipose tissue macrophages to reduce obesity-related inflammation and improve insulin resistance. Int Immunopharmacol. 2024;25(Pt 2):143. 10.1016/j.intimp.2024.113499. 39471690

[25] Rong JX, Qiu Y, Hansen MK, Zhu L, Zhang V, Xie M, et al. Adipose mitochondrial biogenesis is suppressed in db/db and high-fat diet-fed mice and improved by rosiglitazone. Diabetes. 2007;56(7):1751–60. 10.2337/db06-1135. 17456854

[26] Villarroya F, Iglesias R, Giralt M. PPARs in the control of uncoupling proteins gene expression. PPAR Res. 2007;2007:74364. 10.1155/2007/74364. 17389766 PMC1779581

[27] Choo HJ, Kim JH, Kwon OB, Lee CS, Mun JY, Han SS, et al. Mitochondria are impaired in the adipocytes of type 2 diabetic mice. Diabetologia. 2006;49(4):784–91. 10.1007/s00125-006-0170-2. 16501941

[28] Yin X, Lanza IR, Swain JM, Sarr MG, Nair KS, Jensen MD. Adipocyte mitochondrial function is reduced in human obesity independent of fat cell size. J Clin Endocrinol Metab. 2014;99(2):E209–16. 10.1210/jc.2013-3042. 24276464 PMC3913808

[29] Heinonen S, Muniandy M, Buzkova J, Mardinoglu A, Rodríguez A, Frühbeck G, et al. Mitochondria-related transcriptional signature is downregulated in adipocytes in obesity: a study of young healthy MZ twins. Diabetologia. 2017;60(1):169–81. 10.1007/s00125-016-4121-2. 27734103

[30] Maassen JA, Romijn JA, Heine RJ. Fatty acid-induced mitochondrial uncoupling in adipocytes as a key protective factor against insulin resistance and beta cell dysfunction: a new concept in the pathogenesis of obesity-associated type 2 diabetes mellitus. Diabetologia. 2007;50(10):2036–41. 10.1007/s00125-007-0776-z. 17712547 PMC2039833

[31] Chattopadhyay M, Guhathakurta I, Behera P, Ranjan KR, Khanna M, Mukhopadhyay S, et al. Mitochondrial bioenergetics is not impaired in nonobese subjects with type 2 diabetes mellitus. Metabolism. 2011;60(12):1702–10. 10.1016/j.metabol.2011.04.015. 21663924

[32] Vázquez-Vela ME, Torres N, Tovar AR. White adipose tissue as endocrine organ and its role in obesity. Arch Med Res. 2008;39(8):715–28. 10.1016/j.arcmed.2008.09.005. 18996284

[33] MacDougald OA, Burant CF. The rapidly expanding family of adipokines. Cell Metab. 2007;6(3):159–61. 10.1016/j.cmet.2007.08.010. 17767903

[34] Guilherme A, Virbasius JV, Puri V, Czech MP. Adipocyte dysfunctions linking obesity to insulin resistance and type 2 diabetes. Nat Rev Mol Cell Biol. 2008;9(5):367–77. 10.1038/nrm2391. 18401346 PMC2886982

[35] Friedman JM. Leptin and the regulation of body weight. Keio J Med. 2011;60(1):1–9. 10.2302/kjm.60.1. 21460597

[36] Münzberg H, Singh P, Heymsfield SB, Yu S, Morrison CD. Recent advances in understanding the role of leptin in energy homeostasis. F1000Res. 2020;9. 10.12688/f1000research.24260.1. F1000 Faculty Rev PMC7255681 32518627

[37] Frederich RC, Hamann A, Anderson S, Löllmann B, Lowell BB, Flier JS. Leptin levels reflect body lipid content in mice: evidence for diet-induced resistance to leptin action. Nat Med. 1995;1(12):1311–4. 10.1038/nm1295-1311. 7489415

[38] Maffei M, Halaas J, Ravussin E, Pratley RE, Lee GH, Zhang Y, et al. Leptin levels in human and rodent: measurement of plasma leptin and ob RNA in obese and weight-reduced subjects. Nat Med. 1995;1(11):1155–61. 10.1038/nm1195-1155. 7584987

[39] Lee Y, Wang MY, Kakuma T, Wang ZW, Babcock E, McCorkle K, et al. Liporegulation in diet-induced obesity: the antisteatotic role of hyperleptinemia. J Biol Chem. 2001;276(8):5629–35. 10.1074/jbc.M008553200. 11096093

[40] Considine RV, Sinha MK, Heiman ML, Kriauciunas A, Stephens TW, Nyce MR, et al. Serum immunoreactive-leptin concentrations in normal-weight and obese humans. N Engl J Med. 1996;334(5):292–5. 10.1056/NEJM199602013340503. 8532024

[41] Chandran M, Phillips SA, Ciaraldi T, Henry RR. Adiponectin: more than just another fat cell hormone? Diabetes Care. 2003;26(8):2442–50. 10.2337/diacare.26.8.2442. 12882876

[42] Yamauchi T, Kamon J, Waki H, Terauchi Y, Kubota N, Hara K, et al. The fat-derived hormone adiponectin reverses insulin resistance associated with both lipoatrophy and obesity. Nat Med. 2001;7(8):941–6. 10.1038/90984. 11479627

[43] Bullen JW Jr, Bluher S, Kelesidis T, Mantzoros CS. Regulation of adiponectin and its receptors in response to development of diet-induced obesity in mice. Am J Physiol Endocrinol Metab. 2007;292(4):E1079–86. 10.1152/ajpendo.00245.2006. https://journals.physiology.org/doi/full/. 17164441

[44] Singh P, Sharma P, Sahakyan KR, Davison DE, Sert-Kuniyoshi FH, Romero-Corral A, et al. Differential effects of leptin on adiponectin expression with weight gain versus obesity. Int J Obes (Lond). 2016;40(2):266–74. 10.1038/ijo.2015.181. 26374448 PMC4747836

[45] Kern PA, Di Gregorio GB, Lu T, Rassouli N, Ranganathan G. Adiponectin expression from human adipose tissue: relation to obesity, insulin resistance, and tumor necrosis factor-alpha expression. Diabetes. 2003;52(7):1779–85. 10.2337/diabetes.52.7.1779. 12829646

[46] Aguilar-Salinas CA, García EG, Robles L, Riaño D, Ruiz-Gomez DG, García-Ulloa AC, et al. High adiponectin concentrations are associated with the metabolically healthy obese phenotype. J Clin Endocrinol Metab. 2008;93(10):4075–9. 10.1210/jc.2007-2724. 18682512

[47] Schautz B, Later W, Heller M, Müller MJ, Bosy-Westphal A. Associations between breast adipose tissue, body fat distribution and cardiometabolic risk in women: cross-sectional data and weight-loss intervention. Eur J Clin Nutr. 2011;65(7):784–90. 10.1038/ejcn.2011.35. 21427743

[48] Jonas MI, Kurylowicz A, Bartoszewicz Z, Lisik W, Jonas M, Domienik-Karlowicz J, et al. Adiponectin/resistin interplay in serum and in adipose tissue of obese and normal-weight individuals. Diabetol Metab Syndr. 2017;9:95. 10.1186/s13098-017-0293-2. 29213336 PMC5709988

[49] Yang Q, Graham TE, Mody N, Preitner F, Peroni OD, Zabolotny JM, et al. Serum retinol binding protein 4 contributes to insulin resistance in obesity and type 2 diabetes. Nature. 2005;436(7049):356–62. 10.1038/nature03711. 16034410

[50] Graham TE, Yang Q, Blüher M, Hammarstedt A, Ciaraldi TP, Henry RR, et al. Retinol-binding protein 4 and insulin resistance in lean, obese, and diabetic subjects. N Engl J Med. 2006;354(24):2552–63. 10.1056/NEJMoa054862. 16775236

[51] Janke J, Engeli S, Boschmann M, Adams F, Böhnke J, Luft FC, et al. Retinol-binding protein 4 in human obesity. Diabetes. 2006;55(10):2805–10. 10.2337/db06-0616. 17003346

[52] Kovacs P, Geyer M, Berndt J, Klöting N, Graham TE, Böttcher Y, et al. Effects of genetic variation in the human retinol binding protein-4 gene (RBP4) on insulin resistance and fat depot-specific mRNA expression. Diabetes. 2007;56(12):3095–100. 10.2337/db06-1647. 17728376

[53] Bajzová M, Kováčiková M, Vítková M, Klimčáková E, Polák J, Kováčová Z, et al. Retinol-binding protein 4 expression in visceral and subcutaneous fat in human obesity. Physiol Res. 2008;57(6):927–34. 10.33549/physiolres.931379. 18052678

[54] Ribel-Madsen R, Friedrichsen M, Vaag A, Poulsen P. Retinol-binding protein 4 in twins: regulatory mechanisms and impact of circulating and tissue expression levels on insulin secretion and action. Diabetes. 2009;58(1):54–60. 10.2337/db08-1019. 18852328 PMC2606893

[55] Chia CW, Egan JM. Incretins in obesity and diabetes. Ann N Y Acad Sci. 2020;1461(1):104–26. 10.1111/nyas.14211. 31392745 PMC10131087

[56] Aukan MI, Coutinho S, Pedersen SA, Simpson MR, Martins C. Differences in gastrointestinal hormones and appetite ratings between individuals with and without obesity-A systematic review and meta-analysis. Obes Rev. 2023;24(2):e13531. 10.1111/obr.13531. 36416279 PMC10078575

[57] Campbell JE, Drucker DJ. Pharmacology, physiology, and mechanisms of incretin hormone action. Cell Metab. 2013;17(6):819–37. 10.1016/j.cmet.2013.04.008. 23684623

[58] Gasbjerg LS, Bergmann NC, Stensen S, Christensen MB, Rosenkilde MM, Holst JJ, et al. Evaluation of the incretin effect in humans using GIP and GLP-1 receptor antagonists. Peptides. 2020;125:170183. 10.1016/j.peptides.2019.170183. 31693916

[59] Campbell JE, Beaudry JL, Svendsen B, Baggio LL, Gordon AN, Ussher JR, et al. GIPR is predominantly localized to nonadipocyte cell types within white adipose tissue. Diabetes. 2022;71(5):1115–27. 10.2337/db21-1166. 35192688 PMC7612781

[60] Neeland IJ, Marso SP, Ayers CR, Lewis B, Oslica R, Francis W, et al. Effects of liraglutide on visceral and ectopic fat in adults with overweight and obesity at high cardiovascular risk: a randomised, double-blind, placebo-controlled clinical trial. Lancet Diabetes Endocrinol. 2021;9(9):595–605. 10.1016/S2213-8587(21)00179-0. 34358471

[61] Garvey WT, Batterham RL, Bhatta M, Buscemi S, Christensen LN, Frias JP, STEP 5 Study Group, et al. Two-year effects of semaglutide in adults with overweight or obesity: the STEP 5 trial. Nat Med. 2022;28(10):2083–91. 10.1038/s41591-022-02026-4. 36216945 PMC9556320

[62] Rubino DM, Greenway FL, Khalid U, O’Neil PM, Rosenstock J, Sørrig R, et al. STEP 8 Investigators. Effect of weekly subcutaneous semaglutide vs daily liraglutide on body weight in adults with overweight or obesity without diabetes: the STEP 8 randomized clinical trial. JAMA. 2022;327(2):138–50. 10.1001/jama.2021.23619. 35015037 PMC8753508

[63] Aronne LJ. SURMOUNT-4 Investigators. Tirzepatide for maintenance of weight reduction in adults with obesity—reply. JAMA. 2024;331(19):1676. 10.1001/jama.2024.2603. 38630499

[64] Aroda VR, Jørgensen NB, Kumar B, Lingvay I, Laulund AS, Buse JB, et al. trial investigators. High-dose semaglutide (up to 16 mg) in people with type 2 diabetes and overweight or obesity: a randomized, placebo-controlled, phase 2 trial. Diabetes Care. 2025;48(6):905–13. 10.2337/dc24-2425. 40279144 PMC12094194

[65] Grunvald E, Shah R, Hernaez R, Chandar AK, Pickett-Blakely O, Teigen LM, et al. AGA Clinical Guidelines Committee. AGA clinical practice guideline on pharmacological interventions for adults with obesity. Gastroenterology. 2022;163(5):1198–225. 10.1053/j.gastro.2022.08.045. 36273831

[66] Giralt M, Cereijo R, Villarroya F. Adipokines and the endocrine role of adipose tissues. Handb Exp Pharmacol. 2016;233:265–82. 10.1007/164_2015_6. 25903415

[67] Villarroya F, Peyrou M, Giralt M. Adipose tissue, at the core of the action of incretin and glucagon-based anti-obesity drugs. Curr Obes Rep. 2025;14(1):67. 10.1007/s13679-025-00660-w. 40892365 PMC12405374

[68] Ejarque M, Guerrero-Pérez F, de la Morena N, Casajoana A, Virgili N, López-Urdiales R, et al. Role of adipose tissue GLP-1R expression in metabolic improvement after bariatric surgery in patients with type 2 diabetes. Sci Rep. 2019;18(1):6274. 10.1038/s41598-019-42770-1. PMC6472499 31000783

[69] Vendrell J, El Bekay R, Peral B, García-Fuentes E, Megia A, Macias-Gonzalez M, et al. Study of the potential association of adipose tissue GLP-1 receptor with obesity and insulin resistance. Endocrinology. 2011;152(11):4072–9. 10.1210/en.2011-1070. 21862620

[70] Mérida E, Delgado E, Molina LM, Villanueva-Peñacarrillo ML, Valverde I. Presence of glucagon and glucagon-like peptide-1-(7–36) amide receptors in solubilized membranes of human adipose tissue. J Clin Endocrinol Metab. 1993;77(6):1654–7. 10.1210/jcem.77.6.8263154. 8263154

[71] Montrose-Rafizadeh C, Yang H, Wang Y, Roth J, Montrose MH, Adams LG. Novel signal transduction and peptide specificity of glucagon-like peptide receptor in 3T3-L1 adipocytes. J Cell Physiol. 1997;172(3):275–. 10.1002/(SICI)1097-4652(199709)172:3<275::AID-JCP1>3.0.CO;2-L. 83. 9284947

[72] Drucker DJ. The Ascending GLP-1 Road From Clinical Safety to Reduction of Cardiovascular Complications. Diabetes. 2018;67(9):1710–9. 10.2337/dbi18-0008. 30135132

[73] Baudry A, Yang ZZ, Hemmings BA. PKBalpha is required for adipose differentiation of mouse embryonic fibroblasts. J Cell Sci. 2006;119(Pt 5):889–97. 10.1242/jcs.02792. 16478789

[74] Quoyer J, Longuet C, Broca C, Linck N, Costes S, Varin E, et al. GLP-1 mediates antiapoptotic effect by phosphorylating Bad through a beta-arrestin 1-mediated ERK1/2 activation in pancreatic beta-cells. J Biol Chem. 2010;285(3):1989–2002. 10.1074/jbc.M109.067207. 19915011 PMC2804357

[75] Challa TD, Beaton N, Arnold M, Rudofsky G, Langhans W, Wolfrum C. Regulation of adipocyte formation by GLP-1/GLP-1R signaling. J Biol Chem. 2012;287(9):6421–30. 10.1074/jbc.M111.310342. 22207759 PMC3307265

[76] Lavoie H, Gagnon J, Therrien M. ERK signalling: a master regulator of cell behaviour, life and fate. Nat Rev Mol Cell Biol. 2020;21(10):607–32. 10.1038/s41580-020-0255-7. 32576977

[77] Bost F, Aouadi M, Caron L, Binétruy B. The role of MAPKs in adipocyte differentiation and obesity. Biochimie. 2005;87(1):51–6. 10.1016/j.biochi.2004.10.018. 15733737

[78] Lacasa D, Agli B, Giudicelli Y. Zeta PKC in rat preadipocytes: modulation by insulin and serum mitogenic factors and possible role in adipogenesis. Biochem Biophys Res Commun. 1995;217(1):123–30. 10.1006/bbrc.1995.2753. 8526899

[79] Martins FF, Marinho TS, Cardoso LEM, Barbosa-da-Silva S, Souza-Mello V, Aguila MB, et al. Semaglutide (GLP-1 receptor agonist) stimulates browning on subcutaneous fat adipocytes and mitigates inflammation and endoplasmic reticulum stress in visceral fat adipocytes of obese mice. Cell Biochem Funct. 2022;40(8):903–13. 10.1002/cbf.3751. 36169111

[80] Pastel E, McCulloch LJ, Ward R, Joshi S, Gooding KM, Shore AC, et al. GLP-1 analogue-induced weight loss does not improve obesity-induced adipose tissue dysfunction. Clin Sci (Lond). 2017;131(5):343–53. 10.1042/CS20160803. 28049736

[81] Ghanim H, Batra M, Green K, Abuaysheh S, Hejna J, Makdissi A, et al. Liraglutide treatment in overweight and obese patients with type 1 diabetes: a 26-week randomized controlled trial; mechanisms of weight loss. Diabetes Obes Metab. 2020;22(10):1742–52. 10.1111/dom.14090. 32424935

[82] Wegeberg AM, Meldgaard T, Baek A, Drewes AM, Vyberg M, Jessen N, et al. Subcutaneous adipose tissue composition and function are unaffected by liraglutide-induced weight loss in adults with type 1 diabetes. Basic Clin Pharmacol Toxicol. 2021;128(6):773–82. 10.1111/bcpt.13575. 33624417 PMC8251841

[83] García-Vega D, Sánchez-López D, Rodríguez-Carnero G, Villar-Taibo R, Viñuela JE, Lestegás-Soto A, et al. Semaglutide modulates prothrombotic and atherosclerotic mechanisms associated with epicardial fat, neutrophils and endothelial cells network. Cardiovasc Diabetol. 2024;23(1):1. 10.1186/s12933-023-02096-9. 38172989 PMC10765851

[84] Furuhashi M, Saitoh S, Shimamoto K, Miura T. Fatty acid-binding protein 4 (FABP4): pathophysiological insights and potent clinical biomarker of metabolic and cardiovascular diseases. Clin Med Insights Cardiol. 2015;8(Suppl 3):23–33. 10.4137/CMC. 25674026 PMC4315049

[85] Touceda V, Fontana Estevez F, Cacciagiú L, Finocchietto P, Bustos R, Vidal A, et al. Liraglutide improves adipose tissue remodeling and mitochondrial dynamics in a visceral obesity model induced by a high-fat diet. Curr Res Pharmacol Drug Discov. 2024;6:100185. 10.1016/j.crphar.2024.100185. 38846009 PMC11153889

[86] Zhou J, Poudel A, Chandramani-Shivalingappa P, Xu B, Welchko R, Li L. Liraglutide induces beige fat development and promotes mitochondrial function in diet-induced obesity mice partially through AMPK-SIRT-1-PGC1-α signaling pathway. Endocrine. 2019;64(2):271–83. 10.1007/s12020-018-1826-7. 30535743

[87] Zhu E, Yang Y, Zhang J, Li Y, Li C, Chen L, et al. Liraglutide suppresses obesity and induces brown fat-like phenotype via soluble guanylyl cyclase mediated pathway in vivo and in vitro. Oncotarget. 2016;7(49):81077–89. 10.18632/oncotarget.13189. 27835589 PMC5348377

[88] Vaittinen M, Ilha M, Herbers E, Wagner A, Virtanen KA, Pietiläinen KH, et al. Liraglutide demonstrates a therapeutic effect on mitochondrial dysfunction in human SGBS adipocytes in vitro. Diabetes Res Clin Pract. 2023;199:110635. 10.1016/j.diabres.2023.110635. 36958431

[89] Wu J, Boström P, Sparks LM, Ye L, Choi JH, Giang AH, et al. Beige adipocytes are a distinct type of thermogenic fat cell in mouse and human. Cell. 2012;150(2):366–76. 10.1016/j.cell.2012.05.016. 22796012 PMC3402601

[90] Bonnefont JP, Djouadi F, Prip-Buus C, Gobin S, Munnich A, Bastin J. Carnitine palmitoyltransferases 1 and 2: biochemical, molecular and medical aspects. Mol Aspects Med. 2004;25(5–6):495–520. 10.1016/j.mam.2004.06.004. 15363638

[91] Houten SM, Wanders RJ. A general introduction to the biochemistry of mitochondrial fatty acid β-oxidation. J Inherit Metab Dis. 2010;33(5):469–77. 10.1007/s10545-010-9061-2. 20195903 PMC2950079

[92] Nagy HM, Paar M, Heier C, Moustafa T, Hofer P, Haemmerle G, et al. Adipose triglyceride lipase activity is inhibited by long-chain acyl-coenzyme A. Biochim Biophys Acta. 2014;1841(4):588–94. 10.1016/j.bbalip.2014.01.005. 24440819 PMC3988850

[93] Jacobsen JM, Petersen N, Torz L, Gerstenberg MK, Pedersen K, Østergaard S, et al. Housing mice near vs below thermoneutrality affects drug-induced weight loss but does not improve prediction of efficacy in humans. Cell Rep. 2024;43(8):114501. 10.1016/j.celrep.2024.114501. 39067024 PMC11380917

[94] Lyu X, Yan K, Wang X, Xu H, Guo X, Zhu H, et al. A novel anti-obesity mechanism for liraglutide by improving adipose tissue leptin resistance in high-fat diet-fed obese mice. Endocr J. 2022;69(10):1233–44. 10.1507/endocrj.EJ21-0802. 35705299

[95] Chung le T, Hosaka T, Yoshida M, Harada N, Sakaue H, Sakai T, et al. Exendin-4, a GLP-1 receptor agonist, directly induces adiponectin expression through protein kinase A pathway and prevents inflammatory adipokine expression. Biochem Biophys Res Commun. 2009;390(3):613–8. 10.1016/j.bbrc.2009.10.015. 19850014

[96] Abbas NAT, El Salem A. Metformin, sitagliptin, and liraglutide modulate serum retinol-binding protein-4 level and adipocytokine production in type 2 diabetes mellitus rat model. Can J Physiol Pharmacol. 2018;96(12):1226–31. 10.1139/cjpp-2017-0650. 30075088

[97] Singh A, Fernandes JRD, Chhabra G, Krishna A, Banerjee A. Liraglutide modulates adipokine expression during adipogenesis, ameliorating obesity and polycystic ovary syndrome in mice. Endocrine. 2019;64(2):349–66. 10.1007/s12020-019-01891-3. 30904998

[98] Song DH, Getty-Kaushik L, Tseng E, Simon J, Corkey BE, Wolfe MM. Glucose-dependent insulinotropic polypeptide enhances adipocyte development and glucose uptake in part through Akt activation. Gastroenterology. 2007;133(6):1796–805. 10.1053/j.gastro.2007.09.005. 18054552 PMC2185546

[99] Regmi A, Aihara E, Christe ME, Varga G, Beyer TP, Ruan X, et al. Tirzepatide modulates the regulation of adipocyte nutrient metabolism through long-acting activation of the GIP receptor. Cell Metab. 2024;36(7):1534–e15497. 10.1016/j.cmet.2024.05.010. 38878772

[100] Mohammad S, Ramos LS, Buck J, Levin LR, Rubino F, McGraw TE. Gastric inhibitory peptide controls adipose insulin sensitivity via activation of cAMP-response element-binding protein and p110β isoform of phosphatidylinositol 3-kinase. J Biol Chem. 2011;286(50):43062–70. 10.1074/jbc.M111.289009. 22027830 PMC3234864

[101] Zaïmia N, Obeid J, Varrault A, Sabatier J, Broca C, Gilon P, et al. GLP-1 and GIP receptors signal through distinct β-arrestin 2-dependent pathways to regulate pancreatic β cell function. Cell Rep. 2023;42(11):113326. 10.1016/j.celrep.2023.113326. 37897727

[102] Kizilkaya HS, Sørensen KV, Madsen JS, Lindquist P, Douros JD, Bork-Jensen J, et al. Characterization of genetic variants of GIPR reveals a contribution of β-arrestin to metabolic phenotypes. Nat Metab. 2024;6(7):1268–81. 10.1038/s42255-024-01061-4. 38871982 PMC11272584

[103] Willard FS, Douros JD, Gabe MB, Showalter AD, Wainscott DB, Suter TM, et al. Tirzepatide is an imbalanced and biased dual GIP and GLP-1 receptor agonist. JCI Insight. 2020;5(17):e140532. 10.1172/jci.insight.140532. 32730231 PMC7526454

[104] Gasbjerg LS, Rosenkilde MM, Meier JJ, Holst JJ, Knop FK. The importance of glucose-dependent insulinotropic polypeptide receptor activation for the effects of tirzepatide. Diabetes Obes Metab. 2023;25(11):3079–92. 10.1111/dom.15216. 37551549

[105] Kim H, Kim SW, Choi C, Joo S, Kim G, Kim M, et al. Comparative analysis of LJ-4378 and tirzepatide in mouse models of obesity and weight regain. Arch Pharm Res. 2025;48(11–12):1441–59. 10.1007/s12272-025-01575-9. 41199017 PMC12680861

[106] Samms RJ, Christe ME, Collins KA, Pirro V, Droz BA, Holland AK, et al. GIPR agonism mediates weight-independent insulin sensitization by tirzepatide in obese mice. J Clin Invest. 2021;131(12):e146353. 10.1172/JCI146353. 34003802 PMC8203452

[107] Masson W, Lobo M, Nogueira JP, Barbagelata L, Touzas P, Frías JP. Anti-inflammatory effects of tirzepatide: a systematic review and meta-analysis. Rev Endocr Metab Disord. 2025. 10.1007/s11154-025-09991-4. 41032183

[108] Mi L, Li T, Xiang X, Zhou Y, Xiong N, Chen Y, et al. Tirzepatide retention in iWAT with mesoporous polydopamine encapsulation enhances weight loss through leptin receptor signaling. J Adv Res. 2025;S2090–12322500692. 10.1016/j.jare.2025.09.009. PMC13227263 40945849

[109] Herman R, Jensterle M, Horvat S, Lezaic L, Snoj Z, Pusnik I, et al. Effect of tirzepatide-induced weight loss on adipose tissue in obesity: rationale and design of the randomized placebo-controlled TABFAT trial. Trials. 2025;26(1):300. 10.1186/s13063-025-09045-9. 40847412 PMC12374325

[110] Thomas MK, Nikooienejad A, Bray R, Cui X, Wilson J, Duffin K, et al. Dual GIP and GLP-1 receptor agonist tirzepatide improves beta-cell function and insulin sensitivity in type 2 diabetes. J Clin Endocrinol Metab. 2021;106(2):388–96. 10.1210/clinem/dgaa863. 33236115 PMC7823251

[111] Caussy C, Cusi K, Rosenstock J, Bugianesi E, Thomas MK, Tang Y, et al. Relationship between metabolic and histological responses in people with MASH with and without type 2 diabetes: SYNERGY-NASH trial analysis. Diabetes Care. 2025;48(12):2074–83. 10.2337/dc25-1306. 41066427 PMC12635880

[112] Wilson JM, Lin Y, Luo MJ, Considine G, Cox AL, Bowsman LM, et al. Tirzepatide improves cardiovascular risk biomarkers in patients with type 2 diabetes: a post hoc analysis. Diabetes Obes Metab. 2022;24(1):148–53. 10.1111/dom.14553. 34542221 PMC9292792

[113] Abdul-Rahman T, Roy P, Ahmed FK, Mueller-Gomez JL, Sarkar S, Garg N, et al. The power of three: Retatrutide’s role in modern obesity and diabetes therapy. Eur J Pharmacol. 2024;985:177095. 10.1016/j.ejphar.2024.177095. 39515565

[114] Jastreboff AM, Kaplan LM, Frías JP, Wu Q, Du Y, Gurbuz S, et al. Triple-hormone-receptor agonist retatrutide for obesity: a phase 2 trial. N Engl J Med. 2023;389(6):514–26. 10.1056/NEJMoa2301972. 37366315

[115] Li Q, Cheng W, Zhang J, Ran S, Yu H, Zhou W, et al. Multi-omic profiling reveals Retatrutide alleviates adipose tissue fibrosis via metabolic reprogramming and tissue repair. Diabetol Metab Syndr. 2026;18:120. 10.1186/s13098-026-02116-0. 41964043 PMC13214175

[116] Goossens GH, Jocken JWE, Blaak EE. Sexual dimorphism in cardiometabolic health: the role of adipose tissue, muscle and liver. Nat Rev Endocrinol. 2021;17(1):47–66. 10.1038/s41574-020-00431-8. 33173188

[117] Ghanta A, Wilson E, Chao AM. Sex Differences in Obesity and Its Treatment. Curr Psychiatry Rep. 2025;27:278–85. 10.1007/s11920-025-01601-z. 40100584

[118] Sharples AJ, Mahawar K. Systematic review and meta-analysis of randomized controlled trials comparing long-term outcomes of Roux-en-Y gastric bypass and sleeve gastrectomy. Obes Surg. 2020;30:664–72. 10.1007/s11695-019-04235-2. 31724116

[119] Peterli R, Steinert RE, Woelnerhanssen B, Peters T, Christoffel-Courtin C, Gass M, et al. Metabolic and hormonal changes after laparoscopic Roux-en-Y gastric bypass and sleeve gastrectomy. Obes Surg. 2012;22(5):740–8. 10.1007/s11695-012-0622-3. 22354457 PMC3319900

[120] Meek CL, Lewis HB, Reimann F, Gribble FM, Park AJ. The effect of bariatric surgery on gastrointestinal and pancreatic peptide hormones. Peptides. 2016;77:28–37. 10.1016/j.peptides.2015.08.013. 26344355

[121] Lecoutre S, Rebière C, Marcelin G, Clément K. How does bariatric surgery remodel adipose tissue? Ann Endocrinol (Paris). 2024;85(3):175–8. 10.1016/j.ando.2024.05.008. 38871506

[122] Cancello R, Henegar C, Viguerie N, Taleb S, Poitou C, Rouault C, et al. Reduction of macrophage infiltration in adipose tissue after weight loss. Diabetes. 2005;54(8):2277–86. 10.2337/diabetes.54.8.2277. 16046292

[123] Zhang H, Wang Y, Zhang J, Potter BJ, Sowers JR, Zhang C. Bariatric surgery reduces visceral adipose inflammation and improves endothelial function in type 2 diabetic mice. Arterioscler Thromb Vasc Biol. 2011;31(9):2063–9. 10.1161/ATVBAHA.111.225870. 21680898 PMC3158262

[124] Tamez M, Ramos-Barragan V, Mendoza-Lorenzo P, Arrieta-Joffe P, López-Martínez S, Rojano-Rodríguez ME, et al. Adipocyte size and leptin receptor expression after Roux-en-Y gastric bypass. Obes Surg. 2017;27(12):3330–2. 10.1007/s11695-017-2930-0. 28924918

[125] Katsogiannos P, Kamble PG, Boersma GJ, Karlsson FA, Lundkvist P, Sundbom M, et al. Early changes in adipose tissue morphology, gene expression, and metabolism after RYGB in patients with obesity and T2D. J Clin Endocrinol Metab. 2019;104(7):2601–13. 10.1210/jc.2018-02165. 30689903

[126] Liu Y, Aron-Wisnewsky J, Marcelin G, Genser L, Le Naour G, Torcivia A, et al. Accumulation and Changes in Composition of Collagens in Subcutaneous Adipose Tissue After Bariatric Surgery. J Clin Endocrinol Metab. 2016;101(1):293–304. 10.1210/jc.2015-3348. 26583585

[127] Andersson DP, Eriksson Hogling D, Thorell A, Toft E, Qvisth V, Näslund E, et al. Changes in subcutaneous fat cell volume and insulin sensitivity after weight loss. Diabetes Care. 2014;37(7):1831–6. 10.2337/dc13-2395. 24760260

[128] Meyer-Gerspach AC, Peterli R, Moor M, Madörin P, Schötzau A, Nabers D, et al. Quantification of Liver, Subcutaneous, and Visceral Adipose Tissues by MRI Before and After Bariatric Surgery. Obes Surg. 2019;29(9):2795–805. 10.1007/s11695-019-03897-2. 31089967 PMC6713693

[129] Bouchard-Mercier A, de Toro-Martín J, Nadeau M, Lescelleur O, Lebel S, Richard D, et al. Molecular remodeling of adipose tissue is associated with metabolic recovery after weight loss surgery. J Transl Med. 2022;20(1):283. 10.1186/s12967-022-03485-6. 35739539 PMC9219157

[130] Aron-Wisnewsky J, Tordjman J, Poitou C, Darakhshan F, Hugol D, Basdevant A, et al. Human adipose tissue macrophages: m1 and m2 cell surface markers in subcutaneous and omental depots and after weight loss. J Clin Endocrinol Metab. 2009;94(11):4619–23. 10.1210/jc.2009-0925. 19837929

[131] Huang D, Zhang Z, Dong Z, Liu R, Huang J, Xu G. Caloric restriction and Roux-en-Y Gastric Bypass promote white adipose tissue browning in mice. J Endocrinol Invest. 2022;45(1):139–48. 10.1007/s40618-021-01626-0. 34232475

[132] van der Kolk BW, Muniandy M, Kaminska D, Alvarez M, Ko A, Miao Z, et al. Differential Mitochondrial Gene Expression in Adipose Tissue Following Weight Loss Induced by Diet or Bariatric Surgery. J Clin Endocrinol Metab. 2021;106(5):1312–24. 10.1210/clinem/dgab072. 33560372 PMC8063261

[133] Pinho ACO, Lazaro A, Barbosa P, Porter C, Tralhão JG, Carvalho E. Impact of the metabolic disease status in obesity and surgical weight loss on human adipose tissue bioenergetics. J Physiol. 2025;603(9):2583–617. 10.1113/JP286103. 40059366

[134] Stefater MA, Pérez-Tilve D, Chambers AP, Wilson-Pérez HE, Sandoval DA, Berger J, et al. Sleeve gastrectomy induces loss of weight and fat mass in obese rats, but does not affect leptin sensitivity. Gastroenterology. 2010;138(7):2426–36. 10.1053/j.gastro.2010.02.059. 20226189 PMC2883635

[135] Haider DG, Schindler K, Prager G, Bohdjalian A, Luger A, Wolzt M, et al. Serum retinol-binding protein 4 is reduced after weight loss in morbidly obese subjects. J Clin Endocrinol Metab. 2007;92(3):1168–71. 10.1210/jc.2006-1839. 17164313

[136] Bachmayer C, Lammert A, Hasenberg T, Hammes HP. Healthy obese and post bariatric patients - metabolic and vascular patterns. Exp Clin Endocrinol Diabetes. 2013;121(8):483–7. 10.1055/s-0033-1347248. 23765752

[137] Wang X, Huang Y, Gao J, Sun H, Jayachandran M, Qu S. Changes of serum retinol-binding protein 4 associated with improved insulin resistance after laparoscopic sleeve gastrectomy in Chinese obese patients. Diabetol Metab Syndr. 2020;12:7. 10.1186/s13098-019-0511-1. 31956345 PMC6961405

[138] Woelnerhanssen B, Peterli R, Steinert RE, Peters T, Borbély Y, Beglinger C. Effects of postbariatric surgery weight loss on adipokines and metabolic parameters: comparison of laparoscopic Roux-en-Y gastric bypass and laparoscopic sleeve gastrectomy–a prospective randomized trial. Surg Obes Relat Dis. 2011;7(5):561–8. 10.1016/j.soard.2011.01.044 21429816

[139] Askarpour M, Alizadeh S, Hadi A, Symonds ME, Miraghajani M, Sheikhi A, et al. Effect of Bariatric Surgery on the Circulating Level of Adiponectin, Chemerin, Plasminogen Activator Inhibitor-1, Leptin, Resistin, and Visfatin: A Systematic Review and Meta-Analysis. Horm Metab Res. 2020;52(4):207–15. 10.1055/a-1129-6785. 32268422

[140] Mousapour P, Tasdighi E, Khalaj A, et al. Sex disparity in laparoscopic bariatric surgery outcomes: a matched-pair cohort analysis. Sci Rep. 2021;11:12809. 10.1038/s41598-021-92254-4. 34140595 PMC8211818

[141] Hider AM, Bonham A, Carlin A, Finks J, Ghaferi A, Varban O, Ehlers AP. Association of Sex Differences on Weight Loss and Complications Following Bariatric Surgery. J Surg Res. 2024;299:359–65. 10.1016/j.jss.2024.04.050. 38795559 PMC11475018

[142] Laferrère B. Effect of gastric bypass surgery on the incretins. Diabetes Metab. 2009;35(6 Pt 2):513–7. 10.1016/S1262-3636(09)73458-5. 20152736 PMC2842993

[143] Laferrère B. Bariatric surgery and obesity: influence on the incretins. Int J Obes Suppl. 2016;6(Suppl 1):S32–6. 10.1038/ijosup.2016.8. 28685028 PMC5485883

[144] Van der Schueren BJ, Homel P, Alam M, Agenor K, Wang G, Reilly D, et al. Magnitude and variability of the glucagon-like peptide-1 response in patients with type 2 diabetes up to 2 years following gastric bypass surgery. Diabetes Care. 2012;35(1):42–6. 10.2337/dc11-1472. 22124715 PMC3241324

[145] Douros JD, Tong J, D’Alessio DA. The Effects of Bariatric Surgery on Islet Function, Insulin Secretion, and Glucose Control. Endocr Rev. 2019;40(5):1394–423. 10.1210/er.2018-00183. 31241742 PMC6749890

[146] Morinigo R, Moizé V, Musri M, Lacy AM, Navarro S, Marín JL, et al. Glucagon-like peptide-1, peptide YY, hunger, and satiety after gastric bypass surgery in morbidly obese subjects. J Clin Endocrinol Metab. 2006;91(5):1735–40. 10.1210/jc.2005-0904. 16478824

[147] Speck M, Cho YM, Asadi A, Rubino F, Kieffer TJ. Duodenal-jejunal bypass protects GK rats from {beta}-cell loss and aggravation of hyperglycemia and increases enteroendocrine cells coexpressing GIP and GLP-1. Am J Physiol Endocrinol Metab. 2011;300(5):E923–32. 10.1152/ajpendo.00422.2010. 21304061

[148] Li JV, Ashrafian H, Bueter M, Kinross J, Sands C, le Roux CW, et al. Metabolic surgery profoundly influences gut microbial-host metabolic cross-talk. Gut. 2011;60(9):1214–23. 10.1136/gut.2010.234708. 21572120 PMC3677150

[149] Guidone C, Manco M, Valera-Mora E, Iaconelli A, Gniuli D, Mari A, et al. Mechanisms of recovery from type 2 diabetes after malabsorptive bariatric surgery. Diabetes. 2006;55(7):2025–31. 10.2337/db06-0068. 16804072

[150] Clements RH, Gonzalez QH, Long CI, Wittert G, Laws HL. Hormonal changes after Roux-en Y gastric bypass for morbid obesity and the control of type-II diabetes mellitus. Am Surg. 2004;70(1):1–4. discussion 4–5. PMID: 14964537. 14964537

[151] Salinari S, Bertuzzi A, Asnaghi S, Guidone C, Manco M, Mingrone G, et al. First-phase insulin secretion restoration and differential response to glucose load depending on the route of administration in type 2 diabetic subjects after bariatric surgery. Diabetes Care. 2009;32(3):375–80. 10.2337/dc08-1314. 19033407 PMC2646012

[152] Andrade S, Lobato CB, Machado M, Hartmann B, Holst JJ, Almeida RF, et al. GLP-1 and GIP may play a role in long-term weight trajectories after gastric bypass. Front Endocrinol (Lausanne). 2025;16:1624001. 10.3389/fendo.2025.1624001. 40687579 PMC12271881

[153] Sarma S, Palcu P. Weight loss between glucagon-like peptide-1 receptor agonists and bariatric surgery in adults with obesity: A systematic review and meta-analysis. Obes (Silver Spring). 2022;30(11):2111–21. 10.1002/oby.23563. 36321278

[154] West S, Scragg J, Aveyard P, Oke JL, Willis L, Haffner SJP, et al. Weight regain after cessation of medication for weight management: systematic review and meta-analysis. BMJ. 2026;392:e085304. 10.1136/bmj-2025-085304. 41500720 PMC12776922

[155] Sabatella L, Ortega PM, Azcárate VV, Sastre FR, Pagola AU, Ahmed A et al. Comparative efficacy of metabolic/bariatric surgery versus GLP-1 receptor agonists: a network meta-analysis of randomized controlled trials. Obesity (Silver Spring). 2025;1. 10.1002/oby.70100 PMC13032060 41326176

[156] Dicker D, Sagy YW, Ramot N, Battat E, Greenland P, Arbel R, et al. Bariatric Metabolic Surgery vs Glucagon-Like Peptide-1 Receptor Agonists and Mortality. JAMA Netw Open. 2024;7(6):e2415392. 10.1001/jamanetworkopen.2024.15392. 38848064 PMC11161844

[157] Stenberg E, Näslund E. Major adverse cardiovascular events among patients with type-2 diabetes, a nationwide cohort study comparing primary metabolic and bariatric surgery to GLP-1 receptor agonist treatment. Int J Obes (Lond). 2023;47(4):251–6. 10.1038/s41366-023-01254-z. 36670155 PMC10113141

[158] Chen H, Guo X, Long T, Li M. Recurrent weight gain after weight loss induced by lifestyle intervention, metabolic and bariatric surgery, or semaglutide in adults with obesity: a systematic review of randomized controlled trials. Obes Surg. 2026;36(2):792–803. 10.1007/s11695-025-08415-1. 41483046

[159] Duan X, Zhang L, Liao Y, Lin Z, Guo C, Luo S, Wang F, Zou Z, Zeng Z, Chen C, Qiu J. Semaglutide alleviates gut microbiota dysbiosis induced by a high-fat diet. Eur J Pharmacol. 2024;15:969176440. 10.1016/j.ejphar.2024.176440. 38402930

[160] Wang R, Lin Z, He M, Liao Y, Xu Y, Chen C, Duan X, Jiang X, Qiu J. The role of gut microbiota in Tirzepatide-mediated alleviation of high-fat diet-induced obesity. Eur J Pharmacol. 2025;51002177827. 10.1016/j.ejphar.2025.177827. 40516844

[161] Yadav J, Liang T, Qin T, Nathan N, Schwenger KJP, Pickel L, et al. Gut microbiome modified by bariatric surgery improves insulin sensitivity and correlates with increased brown fat activity and energy expenditure. Cell Rep Med. 2023;16(5):101051. 10.1016/j.xcrm.2023.101051. PMC10213984 37196633

[162] Tan YW, Shang M, Davis S, Gananadha S. GLP-1 receptor agonists as an adjunct to bariatric surgery for weight loss and metabolic outcome improvement: a systematic review and meta-analysis. Langenbecks Arch Surg. 2025;410(1):295. 10.1007/s00423-025-03831-4. 41071360 PMC12513976

[163] Murvelashvili N, Xie L, Schellinger JN, Mathew MS, Marroquin EM, Lingvay I, et al. Effectiveness of semaglutide versus liraglutide for treating post-metabolic and bariatric surgery weight recurrence. Obes (Silver Spring). 2023;31(5):1280–9. 10.1002/oby.23736. PMC12398307 36998152

[164] Jamal M, Alhashemi M, Dsouza C, Al-Hassani S, Qasem W, Almazeedi S, et al. Semaglutide and Tirzepatide for the Management of Weight Recurrence After Sleeve Gastrectomy: A Retrospective Cohort Study. Obes Surg. 2024;34(4):1324–32. 10.1007/s11695-024-07137-0. 38430320

[165] Miras AD, Pérez-Pevida B, Aldhwayan M, et al. Adjunctive liraglutide treatment in patients with persistent or recurrent type 2 diabetes after metabolic surgery (GRAVITAS): a randomised, double-blind, placebo-controlled trial. Lancet Diabetes Endocrinol. 2019;7:549–59. 10.1016/s2213-8587(19)30157-3. 31174993

[166] Thakur U, Bhansali A, Gupta R, Rastogi A. Liraglutide augments weight loss after laparoscopic sleeve gastrectomy: a randomised, double-blind, placebo-control study. Obes Surg. 2020. 10.1007/s11695-020-04850-4. 32656729

[167] Elhag W, El Ansari W. Effectiveness and safety of liraglutide in managing inadequate weight loss and weight regain after primary and revisional bariatric surgery: anthropometric and cardiometabolic outcomes. Obes Surg. 2022;32:1005–15. 10.1007/s11695-021-05884-y. 35060021

[168] Lautenbach A, Wernecke M, Huber TB, et al. The potential of semaglutide once-weekly in patients without type 2 diabetes with weight regain or insufficient weight loss after bariatric surgery—a retrospective analysis. Obes Surg. 2022. 10.1007/s11695-022-06211-9. 35879524 PMC9532334

[169] Mok J, Adeleke MO, Brown A, et al. Safety and efficacy of liraglutide, 3.0 mg, once daily vs placebo in patients with poor weight loss following metabolic surgery: the BARI-OPTIMISE randomized clinical trial. JAMA Surg. 2023;158:1003–11. 10.1001/jamasurg.2023.2930. 37494014 PMC10372755

[170] Jensen AB, Renström F, Aczél S, Folie P, Biraima-Steinemann M, Beuschlein F, et al. Efficacy of the glucagon-like peptide-1 receptor agonists liraglutide and semaglutide for the treatment of weight regain after bariatric surgery: a retrospective observational study. Obes Surg. 2023;33(4):1017–25. 10.1007/s11695-023-06484-8. 36765019 PMC9918402

[171] Barrett TS, Hafermann JO, Richards S, LeJeune K, Eid GM. Obesity Treatment With Bariatric Surgery vs GLP-1 Receptor Agonists. JAMA Surg. 2025;160(11):1232–9. 10.1001/jamasurg.2025.3590. 40960852 PMC12444648

[172] Bäckdahl J, Franzén L, Massier L, Li Q, Jalkanen J, Gao H, et al. Spatial mapping reveals human adipocyte subpopulations with distinct sensitivities to insulin. Cell Metab. 2021;33(9):1869–e18826. 10.1016/j.cmet.2021.07.018. 34380013

[173] Heymsfield SB, Aronne LJ, Montgomery P, Klickstein LB, Coleman LA, Dole K, et al. Bimagrumab plus semaglutide alone or in combination for the treatment of obesity: a randomized phase 2 trial. Nat Med. 2026;32(3):869–82. 10.1038/s41591-026-04204-0. 41772149 PMC13004672

